# Rethinking 3R strategies: Digging deeper into *AnimalTestInfo* promotes transparency in in vivo biomedical research

**DOI:** 10.1371/journal.pbio.2003217

**Published:** 2017-12-14

**Authors:** Bettina Bert, Antje Dörendahl, Nora Leich, Julia Vietze, Matthias Steinfath, Justyna Chmielewska, Andreas Hensel, Barbara Grune, Gilbert Schönfelder

**Affiliations:** 1 German Federal Institute for Risk Assessment (BfR), German Centre for the Protection of Laboratory Animals (Bf3R), Berlin, Germany; 2 Charité – Universitätsmedizin Berlin, corporate member of Freie Universität Berlin, Humboldt-Universität zu Berlin, and Berlin Institute of Health, Institute of Clinical Pharmacology and Toxicology, Berlin, Germany; University of Edinburgh, United Kingdom of Great Britain and Northern Ireland

## Abstract

In the European Union (EU), animal welfare is seen as a matter of great importance. However, with respect to animal experimentation, European citizens feel quite uninformed. The European Directive 2010/63/EU for the protection of laboratory animals aims for greater transparency and requires that a comprehensible, nontechnical summary (NTS) of each authorised research project involving animals is published by the respective Member State. However, the NTSs remain sleeping beauties if their contents are not easily and systematically accessible. The German web-based NTS database *AnimalTestInfo* is a unique channel for scientists to communicate their work, and provides the opportunity for large-scale analyses of planned animal studies to inform researchers and the public. For an in-depth meta-analysis, we classified the duly completed NTSs submitted to *AnimalTestInfo* in 2014 and 2015 according to the International Classification of Diseases and Related Health Problems (ICD) system. Indexing the NTSs with ICD codes provided a fine-grained overview of the prospective uses of experimental animals. Using this approach, transparency, especially for highly controversial animal research involving, for example, nonhuman primates, is fostered, as it enables pinpointing the envisaged beneficiary down to the level of the addressed disease. Moreover, research areas with many planned projects involving animals can be specified in detail. The development of 3R (replacement, reduction, and refinement) measures in these research areas may be most efficient, as a large number of experimental animals would benefit from it. Indexing NTSs with ICD codes can support governments and funding agencies in advancing target-oriented funding of 3R research. Data drawn from NTSs can provide a basis for the development, validation, and implementation of directed 3R strategies as well as guidance for rethinking the role of animal research models.

## Author summary

The *AnimalTestInfo* database was developed in Germany to make the nontechnical summaries (NTSs) of animal research studies available in a searchable and easily accessible web-based format. This database helps address requirements stipulated in a European Directive regarding the transparency and publication of animal experiments. In this meta-analysis, we analysed NTSs submitted in 2014 and 2015 to *AnimalTestInfo* and classified them according to the International Classification of Diseases and Related Health Problems (ICD) system. Each NTS was assigned an ICD-10 code based on the envisioned beneficiary of the research project. Based on these codes, we conducted various quantitative analyses. Our results provide a granular overview of the prospective uses of experimental animals and identify areas in need of alternative strategies to help replace, reduce, and refine animal research. We hope this data will inform governments and funding agencies in advancing the integrity and reporting of responsible animal research.

## Introduction

Many citizens, particularly in Europe, see animal welfare as a matter of great importance [1]. At the same time, a majority of Europeans do not feel well informed with respect to animal experimentation [2, 3]. Thus, more detailed information on the purposes of animal experiments and their harms is needed to effectually inform the public. Aimed at improving transparency, the European Directive 2010/63/EU for the protection of laboratory animals [4] requires that researchers provide a nontechnical summary (NTS) for each proposed project, including information on its objectives and potential benefits, expected harm, number of animals, species, and a demonstration of compliance with the requirements of the 3R principle (replace, reduce, and refine) [5]. Competent authorities ensure that the contents of the NTS are accurate and correspond to the project application. Animal numbers provided in NTSs are approximate estimates of the numbers of animals expected to be used over a maximum period of 5 years. However, as part of the project application, researchers have to ascertain, on a statistically sound basis, that the minimum number of animals necessary to obtain reliable results is used (Article 22 Directive 2010/63/EU). Anonymised NTSs of all authorised projects must be published by each Member State of the EU to inform the public about research involving animal experiments without jeopardizing intellectual property and confidential information regarding researchers and institutions.

In Germany, the Federal Institute for Risk Assessment (BfR) has been tasked with publishing NTSs in an appropriate format. The BfR has decided to make NTSs available in a searchable web-based database, *AnimalTestInfo* [6], so that anyone can easily access information about planned and authorised projects involving animals. To our knowledge, this is the first web-based NTS database, and it is currently growing by 2,900 entries per year. Users can browse the database based on experimental purpose, species, number of animals used, year of publication, or any key word. However, the contents deposited in *AnimalTestInfo* offer the opportunity to generate more information for research and the public. Processing and analysing the structured information from the extensive and continuously growing number of NTSs allow us to take a bird’s eye view of research involving animal testing [7] and will contribute to evaluating and improving practices in the field of in vivo biomedical research.

The first goal of our pilot study was to demonstrate that it is possible to comprehensively extract additional information embedded in the NTSs about the objectives and expected benefits of authorised projects using an objective classification system. The second goal was to evaluate whether this information can be used to identify research areas in which the development of directed 3R strategies would be most efficient. At present, valid strategies to specify areas of interest in need of 3R measures (e.g., areas in which many animals are used or severe experiments are performed) and objective criteria to monitor their success are lacking. However, this is important information for researchers when rethinking 3R strategies and for third-party donors to fund 3R-relevant research.

Currently, the recall of relevant information from *AnimalTestInfo* based on key word searches is highly variable, but a complete recall of relevant contents is essential to carry out systematic analyses. While metadata deposited in the database, such as animal numbers and species, can be retrieved in a quantitative manner, scientific details specified in free-text fields may be missed because of linguistic pitfalls. Classification-based searching is a standard solution to support the quantitative recall of contents, which is implemented in patent (e.g., Espacenet [8]) and scientific (e.g., PubMed/Medline [9]) databases. Therefore, in a first approach we classified duly completed NTSs submitted in 2014 (2,328 NTSs) and 2015 (2,970 NTSs) by assigning classification codes of the International Classification of Diseases and Related Health Problems (ICD) system established by the World Health Organization (WHO) [10], based on the beneficiary/target population mentioned in the NTS. We chose the ICD, i.e., the German modification ICD-10-GM-2016, because a preliminary survey of the statements in the NTS fields ‘benefits’ indicated that the majority of projects address specific human diseases. In addition, by indexing with ICD-10 codes, information can be retrieved without the linguistic pitfalls of free-text sections. The ICD has several advantages: it is globally accepted, well thought out, and unambiguous assignment to different diseases is possible (for further benefits of ICD-10, see S2 Text). In addition, free online training tools and supporting tools for the actual classification procedure are available [11–13].

In the present study, we first assessed the percentage of NTSs indicating patients as beneficiaries and allocated ICD-10 codes according to S1 Table. The remaining NTSs were classified according to the indicated beneficiaries. To assess whether it is possible to identify research areas encompassing studies involving a high or low number of animals, we compared the average number of animals per NTS for 9 distinct research areas. As biomedical research involving nonhuman primates is highly controversial, we present the allocation of NTSs authorised in 2015 for the use of nonhuman primates to ICD-10 codes separately.

## Results

### NTS classification based on ICD-10 codes

**Fig 1:**
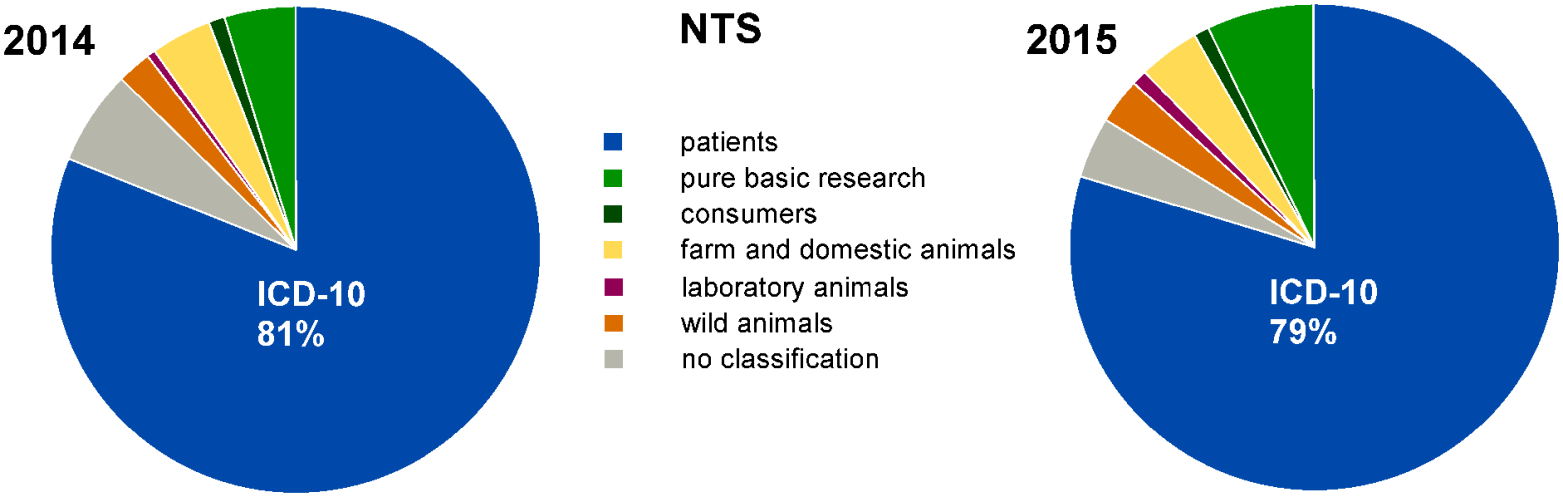
Percentages of target groups for all NTSs. All duly completed NTSs submitted in 2014 (*n* = 2,328) and 2015 (*n* = 2,970) were assigned either to 1 of the 6 target groups or to ‘no classification’. Data are presented as percentages of the total number of duly completed NTSs transferred to us in the respective years. Deviations from 100% are due to mathematical rounding. See also S1 Data and S1 Fig for the corresponding animal numbers. ICD, International Classification of Diseases and Related Health Problems; NTS, nontechnical summary.

First, we determined the percentage of duly completed NTSs submitted in 2014 (*n* = 2,328) and 2015 (*n* = 2,970) that could be assigned an ICD-10-GM-2016 code. Therefore, statements in the fields ‘title’ and ‘benefits’ of each NTS were manually evaluated to determine the beneficiaries. We were able to assign roughly 4/5 of the NTSs, i.e., 81% and 79% in 2014 and 2015, respectively, to an ICD-10 code in the target group ‘patients’ (Fig 1). The remaining NTSs (19% and 21%, respectively) were sorted according to their intended target, i.e., ‘pure basic research’ (5% in 2014 and 7% in 2015), ‘consumers’ (1% each), ‘farm and domestic animals’ (4% each), ‘laboratory animals’ (1% each), and ‘wild animals’ (2% and 3%, respectively), as visualised in Fig 1 (see Materials and methods for definitions). A small percentage of NTSs (6% and 4%, respectively) could not be assigned to a unique target group owing to incomplete or ambiguous statements in the NTS sections describing the project benefits (see Fig 1).

The distribution of numbers of authorised animals included in NTSs is presented in S1 Fig. Notably, of all duly completed NTSs (2,328 in 2014 and 2,970 in 2015), some involved 10,000 or more animals, i.e., 29 NTSs in 2014 and 74 NTSs in 2015. As NTSs applying for 10,000 or more animals were expected to have a substantial impact on the results in analyses of animal numbers, we excluded them from further analyses and evaluated these NTSs separately (see S2 and S3 Figs).

**Fig 2:**
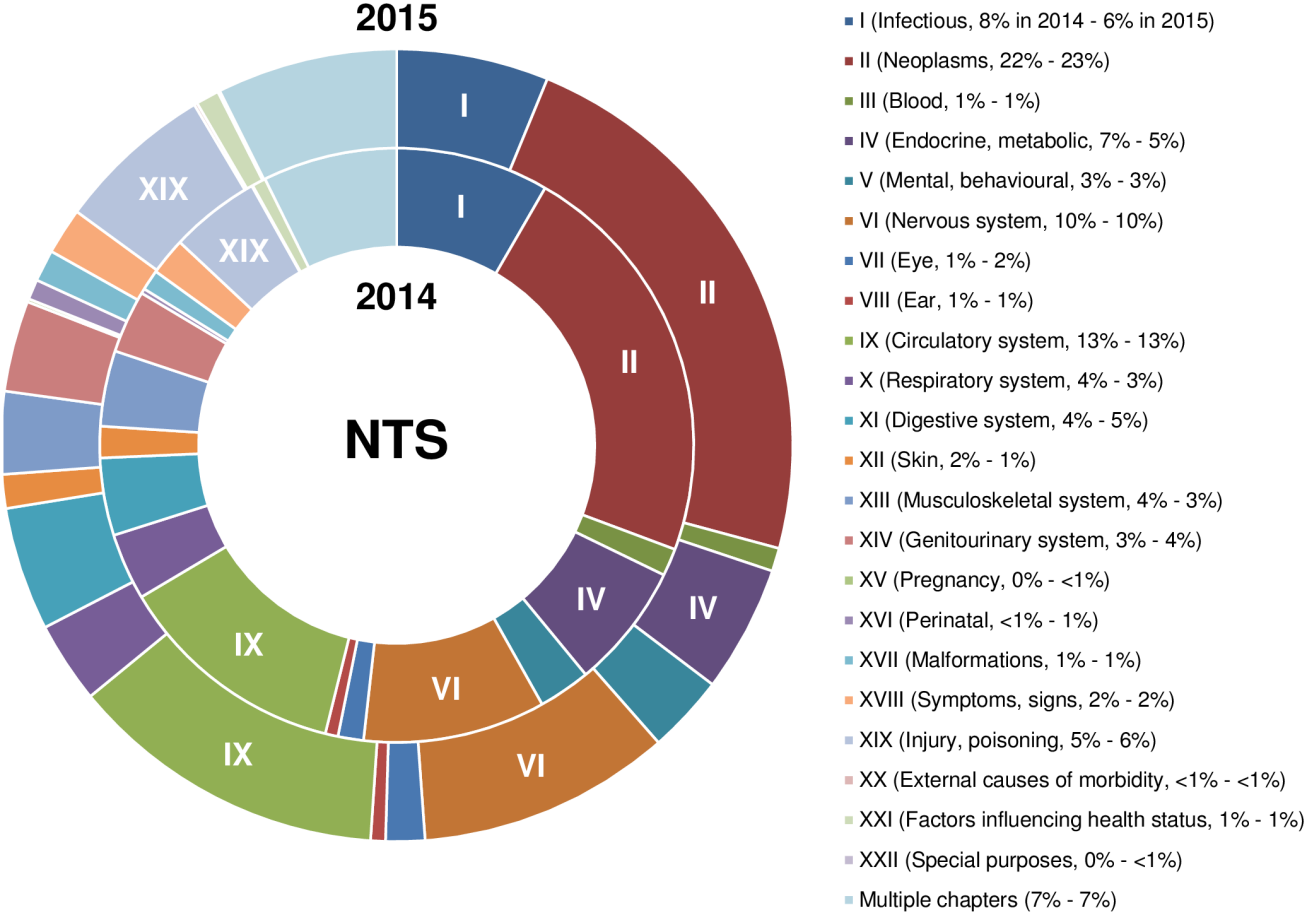
Assignment of NTSs to ICD-10 chapters. The 1,873 NTSs in 2014 (inner ring) and 2,319 in 2015 (outer ring) assigned to the target group ‘patients’ were allocated to 1 of the 22 ICD-10 chapters (abbreviated titles of categories and percentages are indicated in brackets). Note that NTSs involving 10,000 or more animals were excluded; chapter labels are abridgements. Data are presented as percentages of the total number of NTSs of the target group ‘patient’. Deviations from 100% are due to mathematical rounding. See also S1 Data and S4 Fig for the corresponding animal numbers. ICD, International Classification of Diseases and Related Health Problems; NTS, nontechnical summary.

NTSs assigned to the target group ‘patients’ (1,873 NTSs in 2014 and 2,319 NTSs in 2015) were further classified into 1 of the 22 ICD-10 chapters, which yielded a general overview of the proposed and authorised animal experiments. As shown in Fig 2, roughly 2/3 of NTSs assigned to the target group ‘patients’ were distributed across 6 ICD-10 chapters.

The 6 most allocated ICD-10 chapters, in descending order of the number of NTSs assigned, were chapter II, *Neoplasms*, chapter IX, *Diseases of the circulatory system*, chapter VI, *Diseases of the nervous system*, chapter I, *Certain infectious and parasitic diseases*, chapter IV, *Endocrine*, *nutritional and metabolic diseases*, and chapter XIX, *Injury*, *poisoning and certain other consequences of external causes*. The percentages of the remaining ICD-10 chapters varied between <1% (chapter XV, *Pregnancy*, *childbirth and the puerperium*) and 5% (chapter XI, *Diseases of the digestive system*). A low percentage of NTSs in 2014 and 2015 were allocated to more than one ICD-10 chapter (labelled ‘multiple chapters’). It is noteworthy that the proportions of chapters were very similar in both consecutive years, although the number of submitted NTSs increased considerably from 2014 to 2015. The distributions of the corresponding animal numbers are shown in S4 Fig.

**Fig 3:**
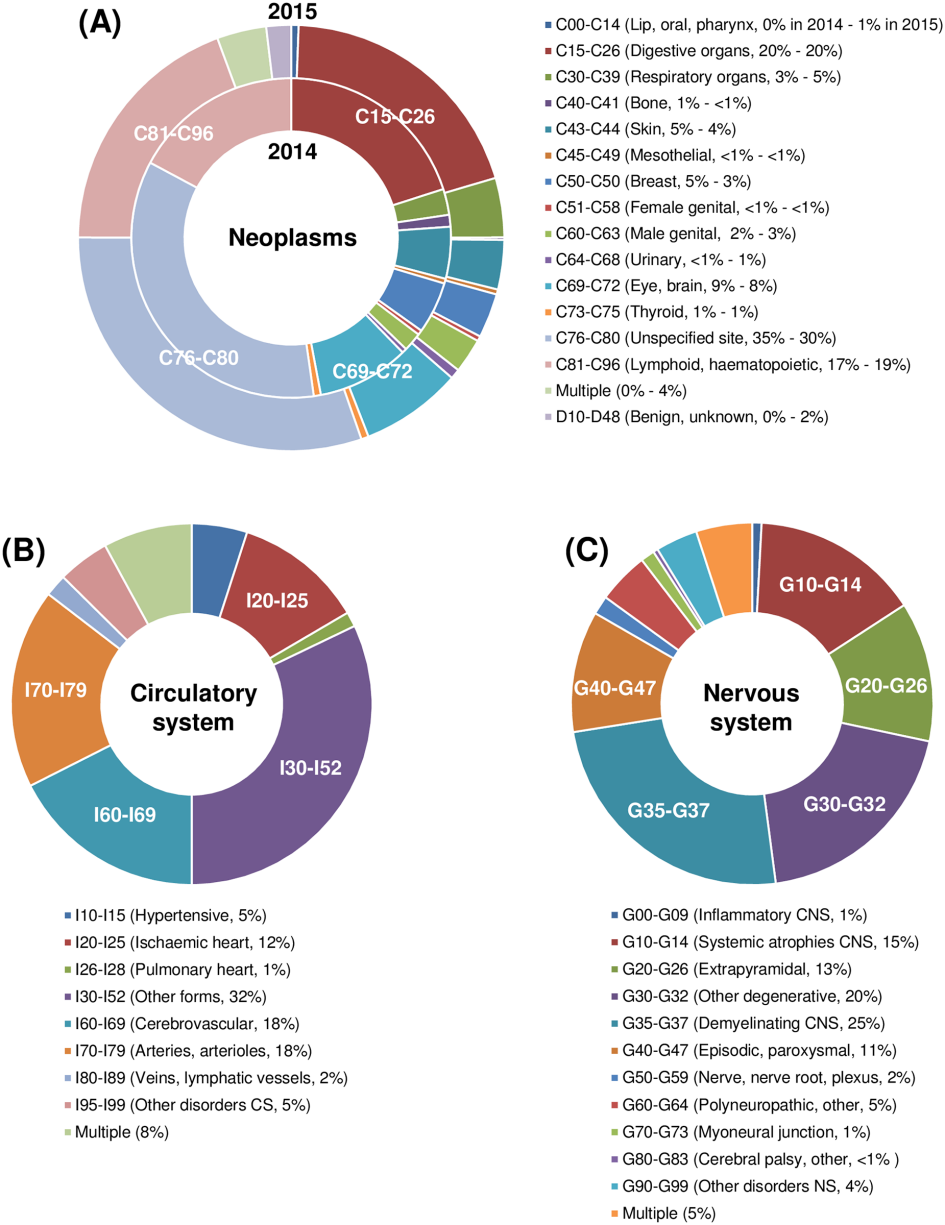
Assignment of NTSs to blocks of 3-character categories for selected ICD-10 chapters. **(A)** For ICD-10 chapter II, *Neoplasms*, 419 NTSs submitted in 2014 and 533 NTSs submitted in 2015, **(B)** for chapter IX, *Diseases of the circulatory system*, 302 NTSs submitted in 2015, and **(C)** for chapter VI, *Diseases of the nervous system*, 240 NTSs submitted in 2015 were allocated to blocks of 3-character categories (abbreviated titles of categories and percentages are indicated in brackets). Data are presented as percentages of the total number of NTSs for the respective chapter and year. Deviations from 100% are due to mathematical rounding. See also S1 Data. CNS, central nervous system; CS, circulatory system; ICD, International Classification of Diseases and Related Health Problems; NS, nervous system; NTS, nontechnical summary; Multiple, assigned to multiple blocks of 3-character categories.

To demonstrate that ICD-10 classification provides a fine-grained picture of envisaged in vivo biomedical research, we further allocated the NTSs to blocks of 3-character categories predefined in the ICD-10 classification. NTSs submitted in 2015 for the 3 most frequent ICD-10 chapters, i.e., chapter II, *Neoplasms*, chapter IX, *Diseases of the circulatory system*, and chapter VI, *Diseases of the nervous system*, were used as an example (Fig 3). As the largest group, NTSs attributed to chapter II, *Neoplasms*, were also analysed for 2014 to assess whether the distribution was constant over time (Fig 3A).

Within the dominantly attributed research area *Neoplasms*, the highest percentage of NTSs in both years was assigned to category C76–C80, *Malignant neoplasms of ill-defined*, *secondary and unspecified sites*, followed by categories C15–C26, *Malignant neoplasms of digestive organs*, C81–C96, *Malignant neoplasms*, *stated or presumed to be primary*, *of lymphoid*, *haematopoietic and related tissue*, and C69–C72, *Malignant neoplasms of eye*, *brain and other parts of central nervous system* (see Fig 3A). Two categories, C00–C14, *Malignant neoplasms of lip*, *oral cavity and pharynx*, and D10–D48, *Benign neoplasms and neoplasms of uncertain or unknown behaviour*, as well as *Multiple* were assigned only to NTSs submitted in 2015, despite that the distribution of NTSs over the blocks of 3-character categories was highly congruent between years.

Within the research area *Diseases of the circulatory system*, nearly 1/3 of NTSs were allocated to the category I30–I52, *Other forms of heart disease*, followed by the categories I60–I69, *Cerebrovascular diseases*, I70–I79, *Diseases of arteries*, *arterioles and capillaries*, and I20–I25, *Ischemic heart diseases* (Fig 3B).

Within the research area *Diseases of the nervous system* (Fig 3C), 1/4 of NTSs were assigned to the category G35–G37, *Demyelinating diseases of the central nervous system*. The second largest group belonged to G30–G32, *Other degenerative diseases of the nervous system*, followed by the categories G10–G14, *Systemic atrophies primarily affecting the central nervous system*, and G40–G47, *Episodic and paroxysmal disorders*.

### Number of animals per NTS

**Fig 4:**
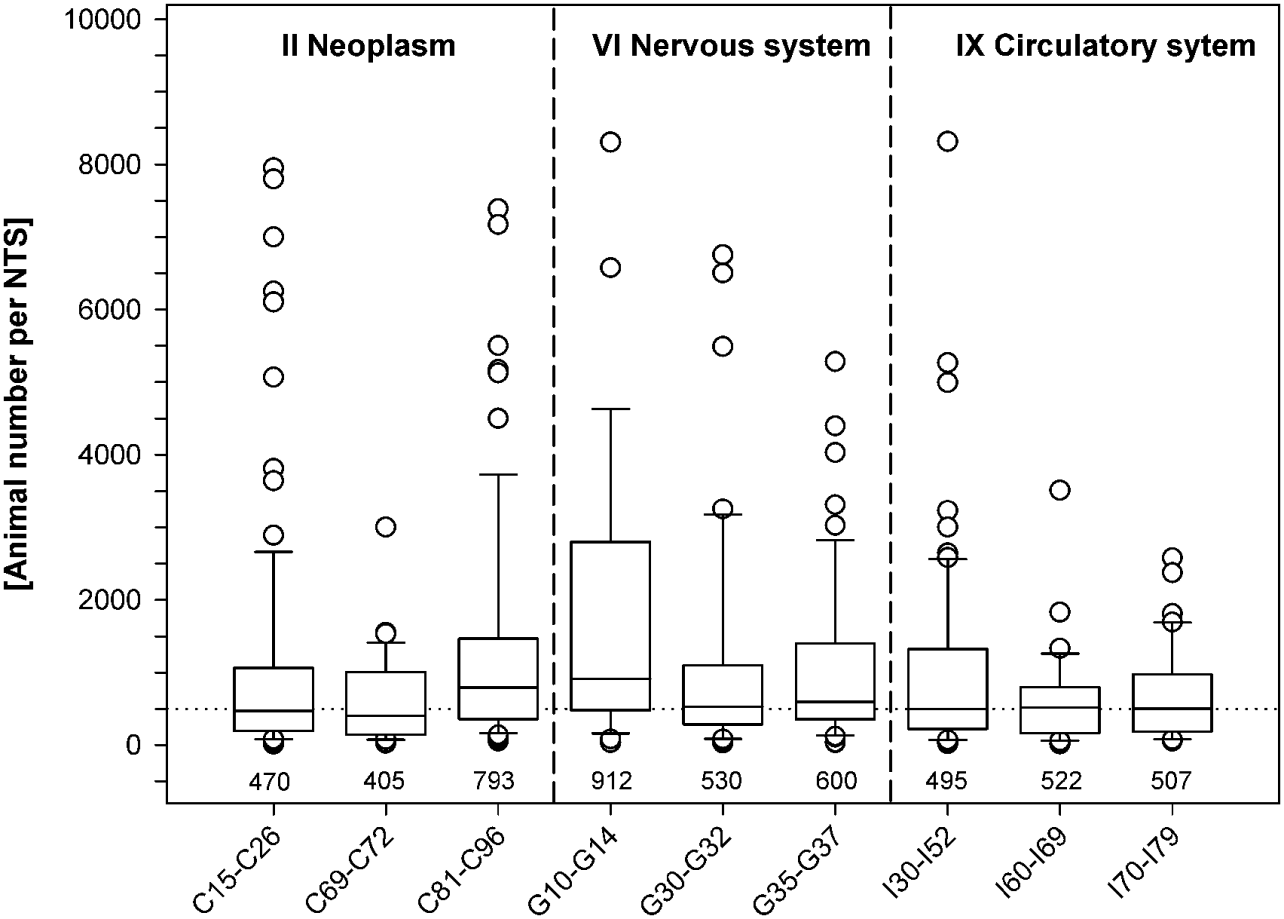
Median animal numbers per NTS for 9 ICD-10 blocks of 3-character categories. Animal numbers were derived solely from NTSs that mention mouse experiments. Data are presented as box plots (medians [25th/75th percentiles], with outliers). The median [animals per NTS] for each category is indicated below the respective box plot. The dotted line refers to *n* = 500 animals per NTS. C15–C26, *Malignant neoplasms of digestive organs* (number of NTSs, *n* = 97); C69–C72, *Malignant neoplasms of eye*, *brain and other parts of central nervous system* (*n* = 38); C81–C96, *Malignant neoplasms*, *stated or presumed to be primary*, *of lymphoid*, *haematopoietic and related tissue* (*n* = 78); G10–G14, *Systemic atrophies primarily affecting the central nervous system* (*n* = 29); G30–G32, *Other degenerative diseases of the nervous system* (*n* = 40); G35–G37, *Demyelinating diseases of the central nervous system* (*n* = 53); I30–I52, *Other forms of heart disease* (*n* = 70); I60–I69, *Cerebrovascular diseases* (*n* = 39); I70–I79, *Diseases of arteries*, *arterioles and capillaries* (*n* = 41). See also S1 Data. ICD, International Classification of Diseases and Related Health Problems; NTS, nontechnical summary.

To assess whether it is possible to specify research areas for projects requiring animal numbers above or below the average, we compared, as an example, the median numbers of animals per NTS for 9 research areas chosen from ICD-10 chapters II, *Neoplasms*, IX, *Diseases of the circulatory system*, and VI, *Diseases of the nervous system* (see Fig 4). As species could be a confounding factor for discrepancies between the proportions of NTSs and animal numbers, we only analysed those NTSs that mentioned the use of mice as laboratory animal species.

The median values ranged from 405 animals per NTS for category C69–C72 to 912 animals per NTS for category G10–G14. There was a significant difference in the distribution of animal numbers per NTS among the 9 ICD-10 blocks of 3-character categories (Kruskal–Wallis test, *p* = 0.0026). Subsequent analysis showed that for C81-C96, *Malignant neoplasms*, *stated or presumed to be primary*, *of lymphoid*, *haematopoietic and related tissue* (versus C15–C26, C69–C72, I60–I69, and I70–I79), G10–G14, *Systemic atrophies primarily affecting the central nervous system* (versus C15–C26, C69–C72, I30–I52, I60–I69, and I70–I79), and G35–G37, *Demyelinating diseases of the central nervous system* (versus C69–C72 und I60–I69), the median animal numbers specified in the NTSs were significantly higher (pairwise Wilcoxon rank sum tests, *p* < 0.05). Notably, despite the thematic differences, the medians for the remaining 6 categories were approximately 500 animals per NTS (Fig 4).

### Analysis of NTSs involving nonhuman primates

Biomedical research involving nonhuman primates is one of the most controversial areas, and analysing NTSs can improve transparency. Hence, we analysed the statements described in the benefit section of 68 NTSs for projects authorised in 2015 for the use of nonhuman primates (total number of animals = 4,024).

The majority of projects involving nonhuman primates could be allocated to the target group ‘patients’ (56 NTSs), while a small fraction (4 NTSs) refers to the target group ‘pure basic research’. Most projects were intended for use in late preclinical testing, as 47 out of 68 NTSs mention ‘toxicity’, ‘safety’, or ‘pharmacodynamics’/’pharmacokinetics’ in their titles. However, 8 NTSs (accounting for 40% of animals) could not be assigned an ICD-10 code or be attributed to one of the other target groups (see Table 1). This includes a single NTS authorised for the use of 1,440 cynomolgus monkeys that describes late-preclinical toxicity studies but does not state a specific disease in the benefit field.

**Table 1:** Target group distribution for nonhuman primates.

Target group	Cynomolgus monkeys	Rhesus monkeys	Marmosets and tamarins	Prosimians	Vervet monkeys	Other nonhuman primates	Total	Percent
**Patient**	1,873	120	182				**2,175**	**54**
**Pure basic research**		35	67	140			**242**	**6**
**No classification**	1,556	16			25	10	**1,607**	**40**
**Total**							**4,024**	

All nonhuman primates of NTSs submitted in 2015 (total animals = 4,024) were allocated to target groups. Data are shown as the absolute animal numbers for each nonhuman primate species and as percentages of all nonhuman primates.

**Abbreviation**: NTS, nontechnical summary.

Nonhuman primates in NTSs assigned to the target group ‘patients’ comprised 3,429 cynomolgus monkeys, 233 rhesus monkeys, and 187 marmosets/tamarins, which were further analysed according to the associated ICD-10 code (see Table 2). The dominant ICD-10 chapters were, in descending order of the numbers of animals involved, chapter II, *Neoplasms*, and chapter VI, *Diseases of the nervous system*, which corresponds to the ranking of ICD-10 chapters attributed to all animals of NTSs submitted in 2015. Notably, a high percentage of nonhuman primates were intended to be used for research in the area *Diseases of the musculoskeletal system and connective tissue* (chapter XIII). Additionally, a large number of nonhuman primates were authorised for research and testing covered by multiple ICD-10 chapters. Our analysis also showed that cynomolgus monkeys are broadly used in various research areas, as they are represented in all ICD-10 chapters listed in Table 2 except for chapter X, *Diseases of the respiratory system*. The assignment of nonhuman primates to blocks of 3-character categories is shown in Table 2.

**Table 2:** Assignment of nonhuman primates to blocks of 3-character categories.

ICD-10 classification			Cynomolgus monkeys	Rhesus monkeys	Marmosets and tamarins	Total	Percentage of target group ‘patients’
Chapter	Number	Blocks of 3-character category^1^					
*Certain infectious and parasitic diseases*	**I**	B15–B19	46	-	-	**46**	**7**
		B20–B24	-	32	-	**32**	
		B95–B98	-	78	-	**78**	
*Neoplasms*	**II**	C15–C26	130	-	-	**130**	**29**
		C60–C63	30	-	-	**30**	
		C69–C72	78	-	-	**78**	
		C76–C80	126	9	-	**135**	
		C81–C96	152	-	-	**152**	
		multiple	100	-	-	**100**	
*Endocrine*, *nutritional*, *and metabolic disorders*	**IV**	E65–E68	15	-	-	**15**	**1**
*Mental and behavioural disorders*	**V**	F00–F09	54	-	-	**54**	**4**
		F20–F29	-	6	-	**6**	
		F80–F89	-	-	21	**21**	
		F00–F99^2^	2	-	-	**2**	
*Diseases of the nervous system*	**VI**	G10–G14	234	-	-	**234**	**17**
		G20–G26	6	-	18	**24**	
		G30–G32	61	-	-	**61**	
		G35–G37	40	-	-	**40**	
		multiple	-	-	6	**6**	
*Diseases of the eye and adnexa*	**VII**	H40–H42	10	-	-	**10**	**<1**
*Diseases of the circulatory system*	**IX**	I20–I25	21	-	-	**21**	**2**
		I30–I52	-	25	-	**25**	
*Diseases of the respiratory system*	**X**	J40–J47	-	-	50	**50**	**2**
*Diseases of the musculoskeletal system and connective tissue*	**XIII**	M00–M03	66	-	10	**76**	**13**
		M05–M14	96	-	-	**96**	
		M30–M36	70	-	-	**70**	
		multiple	36	-	-	**36**	
*Diseases of the genitourinary system*	**XIV**	N30–N39	43	-	-	**43**	**2**
*Multiple chapters*			457	32	15	**504**	**23**
**Total**			**1,873**	**120**	**182**	**2,175**	

All nonhuman primates of NTSs submitted in 2015 (*n* = 4,024) were allocated to ICD-10 chapters and blocks-of-3-characters categories. Data are presented as the total number of animals and, with respect to the ICD-10 chapters, as the percentages of all animals assigned to the target group ‘patients’. Deviations from 100% are due to mathematical rounding.

^1^B15–B19, *Viral hepatitis*; B20–B24, *Human immunodeficiency virus (HIV) disease*; B95–B98, *Bacterial*, *viral and other infectious agents*; C15–C26, *M*.*n*. *of digestive organs*; C60–C63, *M*.*n*. *of male genital organs*; C69–C72, *M*.*n*. *of eye*, *brain and other parts central nervous system*; C76–C80, *M*.*n*., *secondary and ill-defined*; C81–C96, *M*.*n*., *stated or presumed to be primary*, *of lymphoid*, *haematopoietic and related tissue*; E65–E68, *Obesity and other hyperalimentation*; F00–-F09, *Organic*, *including symptomatic*, *mental disorders*; F20–F29, *Schizophrenia*, *schizotypal and delusional disorders*; F80–F89, *Disorders of psychological development*; F00–F99, *Mental and behavioural disorders*^2^; G10–G14, *Systemic atrophies primarily affecting the central nervous system*; G20–G26, *Extrapyramidal and movement disorders*; G30–G32, *Other degenerative diseases of the nervous system*; G35–G37, *Demyelinating diseases of the central nervous system*; H40–H42, *Glaucoma*; I20–I25, *Ischemic heart diseases*; I30–I52, *Other forms of heart disease*; J40–J47, *Chronic lower respiratory diseases*; M00–M03, *Infectious arthropathies*; M05–M14, *Inflammatory polyarthropathies*; M30–M36, *Systemic connective tissue disorders*; N30–N39, *Other diseases of urinary system*.

^2^Only allocation to the ICD-10 chapter was possible.

**Abbreviations**: M.n., Malignant neoplasms; NTS, nontechnical summary.

## Discussion

Using data available in *AnimalTestInfo*, we were able to demonstrate that systematic analyses of NTSs can increase transparency with regard to the objectives and expected benefits of upcoming animal research. In addition, we were able to specify research areas involving large numbers of in vivo studies, in which the development of targeted 3R measures may be particularly beneficial.

### Increasing transparency in animal research

In the overwhelming majority of cases, the envisaged beneficiaries indicated by researchers in the free-text fields of NTSs are patients. Approximately 80% of the NTSs analysed in this study were assigned to an ICD-10 code, and the percentage of NTSs in each ICD-10 category remained largely stable over 2 consecutive years. Thus, the largest proportion of animal experiments seems to be driven by a human disease-based objective, regardless of the indicated purpose of the authorised project, e.g., ‘basic research’ or ‘translational/applied research’.

NTS allocation to the 22 ICD-10 chapters can give a prospective, in-depth picture of the objectives of animal experiments. Current legal regulations prescribe that the use of experimental animals is retrospectively reported in detail but differentiate only 12 purposes, all related to human diseases [14]. Our study revealed that NTSs were distributed over all 22 ICD-10 chapters, reflecting the broad spectrum of current research activities in Germany. The 6 most frequently attributed ICD-10 chapters were II, *Neoplasms*, IX, *Diseases of the circulatory system*, VI, *Diseases of the nervous system*, I, *Certain infectious and parasitic diseases*, IV, *Endocrine*, *nutritional and metabolic diseases*, and XIX, *Injury*, *poisoning and certain other consequences of external causes*. Remarkably, these 6 research areas are also target areas of the German federal government’s health programme. This programme supports research efforts to combat the 6 most common diseases occurring in the German population [15]. It is not surprising that there is great overlap between the most attributed ICD-10 chapters in our analysis and the research areas represented by the ‘German Centres for Health Research’. Moreover, the 6 research areas addressed by most NTSs are in line with the most common diseases mentioned in the recent health report published by the Robert Koch-Institute [16]. The report lists the 10 main causes of death according to their ICD-10 codes, referring to 6 chapters, 5 of which were also among the 6 most frequently attributed ICD-10 chapters in our analysis. This high concordance supports the validity of NTS allocation to ICD-10 chapters in this pilot study, suggesting that the analysis can provide guidance for the development and support of directed 3R measures for specific research areas. The implementation of the 3R principle could be strengthened within the scientific landscape by establishing 3R ‘hubs’ in the German Centres for Health Research. The infrastructure and concentrated scientific competence of those centres enables the efficient development of specific alternative methods for the 6 research areas that are most highly represented in the NTSs. A major advantage of this strategy is that scientists working with animals can directly interact with those developing alternative methods. This translational scientific strategy could help to identify the best models to accurately mimic the human disease condition.

Allocation of NTSs to ICD-10 codes at the ‘block of 3-character category’ level, as shown for the 3 chapters *Neoplasms*, *Diseases of the circulatory system*, and *Diseases of the nervous system*, allows a prospective, more fine-grained overview of the intended benefits of in vivo biomedical research than the retrospective overviews requested by European regulations [14]. For example, within the field of cancer research the annual statistical reports about animal usage defined by the European Commission only differentiate between the categories ‘oncology’ for basic research and ‘human cancer’ for translational and applied research [14]. With the ICD-10, we were able to distinguish 15 types of neoplasms, according to the affected organ sites, for planned animal research projects. Within the research area *Neoplasms*, about 2/3 of the NTSs described studies of cancers of specified sites, i.e., *digestive organs*, *eye*, *brain and central nervous system*, *breast*, and *skin* or of cancers of *lymphoid*, *haematopoietic system and related tissues*. Notably, with a difference of 1%–2%, the distribution of research areas within the field of *Neoplasms* was highly congruent between 2014 and 2015. Comparing our results with the latest ‘Cancer in Germany’ report published by the Robert Koch-Institute in 2016 [17], the distribution of planned in vivo projects related to different cancer types is highly congruent with the morbidity and death rates of the respective form of cancer. For example, *malignant neoplasms of digestive organs* (C15–C26) are mostly allocated to NTSs in the field of neoplasms and are also linked to high morbidity and death rates [17]. Notably, some types of cancers, including skin, breast, prostate, and lung cancers, are not a focus of in vivo cancer research, as the high morbidity and death rates might suggest, and in view of these forms of cancer being highly relevant from a global point of view [18]. Other forms of cancer, involving the lymphoid; haematopoietic system and related tissues; and eye, brain, and central nervous system, rank high with respect to in vivo research but affect a relatively small proportion of the population [17]. A possible explanation for this discrepancy is that in certain areas of preclinical cancer research, sound in vitro or ex vivo models are available, although their validity is controversial [19, 20]. Furthermore, our analysis showed that 1/3 of NTSs refer to unspecified neoplasms, i.e., *Malignant neoplasms of ill-defined*, *secondary and unspecified sites* (C76–C80), and thus their proposed specific benefit is unclear. NTSs that mention ‘cancer research’ without further differentiation were allocated to this category. Here, the quality of NTSs and, consequently, that of the analysis thereof, could be increased if researchers provided a more precise description of the area of research, thereby supporting the identification of other key aspects for the development of alternative methods according to the 3R principle.

This deeper analysis of NTSs indexed with ICD-10 codes suggests that a substantial amount of experimental animal research, including basic research, is driven by a human disease-based approach focusing on diseases with global relevance. Notwithstanding this, there is an urgent necessity for research in fields that affect only a small group of the population but have a detrimental effect on the quality of life of those concerned, and for research in other areas, such as pure basic research that aims to advance knowledge without direct applications to practical problems in the near future.

Indexing NTSs with ICD-10 codes can also support transparency in research areas that are highly controversial, for instance, those experiments involving nonhuman primates [21]. Respecting public interest, Directive 2010/63/EU stipulates in Article 42 that all research projects using nonhuman primates have to be authorised by the competent authority, irrespective of whether they apply for a simplified procedure necessary to satisfy regulatory requirements [4]. Thereby, the public is informed of all planned projects using nonhuman primates by corresponding NTSs. The ICD-10 classification of NTSs provides fine-scale information about the prospective benefits of authorised nonhuman primate experiments beyond the current reporting of numbers of animals, as recently analysed for the United Kingdom [22]. More than half of the nonhuman primates for which applications were submitted in Germany in 2015 addressed specified human diseases. The envisaged benefits were associated with 10 ICD-10 chapters, and the largest fraction was related to chapter II, *Neoplasms*, followed by chapters VI, *Diseases of the nervous system*, XIII, *Diseases of the musculoskeletal system and connective tissue*, and I, *Certain infectious and parasitic diseases*. Compared to the allocation of all animals to ICD-10 chapters, as depicted in S4 Fig, the variety of research using nonhuman primates is narrower. In particular, a high number of nonhuman primates were included in research projects focused on *Diseases of the musculoskeletal system and connective tissue* (chapter XIII), and this chapter ranked 11th in 2015 when looking at all animal species. Interestingly, for nonhuman primates, human musculoskeletal disorders were not represented in the German statistics on the reporting of experimental animals in 2014 or 2015. This observation might indicate an increasing trend in research in this area. However, as authorised animal projects can be conducted within a period of 5 years, it is possible that this research activity will not appear in the current and next statistical reports.

A small number of NTSs referring to a large number of nonhuman primates could not be classified, owing to vague language in the benefit and title fields. This is caused, in particular, by a single NTS for a large toxicity study using cynomolgus monkeys. Although the respective project obviously addresses the target group ‘patients’, we were unable to allocate this specific NTS with an ICD-10 code. If the applicant of this particular NTS provided a more specific indication of the beneficiary, enabling the assignment of an ICD-10 code, the percentage of nonhuman primate research relevant to human diseases might have increased to approximately 95%. This example suggests that, especially in the field of nonhuman primate research, applicants often do not provide detailed information about their project in the NTS, either to safeguard their intellectual property or to protect their anonymity. Asking researchers to indicate the most relevant ICD-10 code when entering information about their project in the *AnimalTestInfo* database is a practicable solution to this problem. One of our next steps, therefore, is to integrate the ICD-10 classification system in *AnimalTestInfo*. Researchers then can more precisely and consistently describe the benefits of their project on a voluntary basis, which would substantially improve the quality of NTSs.

### Identification of research areas to promote 3R measures

Despite increasing transparency in animal research, our analysis using the ICD-10 system uniquely provides the scientific community with detailed and data-based information about potential research areas in which the promotion of 3R measures would be most efficient.

Currently, research on alternatives to animal experiments and other 3R measures is often conducted randomly, without specifying concrete targets, i.e., without naming those research areas that will benefit from the respective 3R strategy. Approaches that are not inspired by the reality of in vivo research, however, risk overlooking crucial needs of the prospective users, which are pivotal for later acceptance. We are now able to precisely identify research areas that consistently include a multiple number of in vivo projects, such as *Malignant neoplasms of digestive organs* (C15–C26), *Cerebrovascular diseases* (I60–I69), and *Demyelinating diseases of the central nervous system* (G35–G37). This information can be used by third-party funders as well as by researchers to develop, validate, and implement targeted 3R measures. We postulate that the development of 3R strategies for research areas with many NTSs would be most efficient, as a large number of animals would benefit, irrespective of whether replacement, reduction, or refinement is addressed.

Another unique feature of indexing NTS with ICD-10 codes is the possibility to plot animal numbers retrieved from single projects against the associated ICD-10 code of a specific human disease. As an example, we calculated the average number of animals per NTS for 9 specific research areas within the 3 main research fields *Neoplasms*, *Diseases of the circulatory system*, and *Diseases of the nervous system* for mouse studies. The median number of animals was approximately 500 per NTS. Nonetheless, 2 research areas seemed to require more animals than average, i.e., the category *Lymphoid*, *haematopoietic cancers*, requiring about 800 animals per NTS, and the category *Systemic atrophies primarily affecting the central nervous system*, requiring about 900 animals per NTS. There are many potential explanations for these large requested animal numbers. It is possible that some experimental settings need more animals than others, e.g., due to high variability of the chosen end points or weak effects of interventions. Indeed, meta-analyses have shown high variation in the magnitude of standardised effects among different areas of research. For instance, Holman et al. [23] found standardised effect strengths of 0.84 for cancer and 1.42 for stroke, leading to group sizes of 24 and 9 animals, respectively, to verify statistically significant differences. Thus, a high number of animals per NTS could be the result of a sound sample size calculation to ensure adequate statistical power [24]. Low statistical power contributes to an inability to detect true effects and to the nonreproducibility of study results. Additionally, the positive predictive value diminishes with decreasing power. Therefore, preliminary power analyses are recommended to save animal lives in the long run.

Another explanation for differences in the number of animals per NTS is that early preclinical studies (i.e., exploratory studies) might require more animals than late preclinical studies, such as evaluations of effectiveness, safety, or toxicity (confirmatory studies). The relevance of exploratory studies may depend on the specific area of research. Other plausible explanations for relative high animal numbers per NTS are the merging of several separate projects into a single large project or projects authorised for the breeding and maintenance of genetically altered animals. Thus, knowledge of these potential factors is indispensable to conduct a quantitative evaluation of authorised animal numbers and to support potential 3R measures. Due to their simplicity, the contents of NTSs, however, do not allow for more detailed analyses of why some research areas deviate with respect to animal numbers. Such analysis could only be carried out if the contents of the whole project application for animal experiments are available or by means of an animal study registry, which recent studies have called for [25, 26]. Still, we believe that these figures can provide impetus for analyses of the underlying causes and may encourage the development of appropriate 3R measures, not only for replacement and reduction but also for refinement, as a large number of animals would benefit, irrespective of causal factors.

In the coming years, we aim to use the information on NTS numbers and average animal numbers per NTS to provide researchers with an ‘NTS map’ of in vivo biomedical research, depicting trends in planned animal experiments. This will enable researchers to formulate hypotheses for increased or decreased animal usage observed in specific research areas identified by ICD-10 codes and to define potential 3R strategies. The success of replacement or reduction measures could subsequently be monitored by means of the map, as a peak should diminish over time. We realise that data for more than 2 years are needed to distinguish between a decrease in animal use caused by successful reduction or replacement measures from normal fluctuations, but we are convinced that this study provides a basis for a data-based navigation system to develop targeted 3R strategies and to measure the success of alternative methods. One limitation that needs to be addressed is related to NTSs that involve large numbers of animals. In our present study, a low percentage of NTSs involved a large number of animals, defined as 10,000 animals or more, i.e., 30% and 50% of the total animals in 2014 and 2015, respectively. As a large percentage of these NTSs could not be attributed to any target group, a lot of information concerning their benefits is missing, which becomes relevant when animal numbers are analysed (see S1 Text).

### Conclusions

The present pilot study shows that NTSs contain crucial information on animal research that has not yet been considered and goes beyond the intent of the Directive 2010/63/EU to purely inform the public about animal experiments. Continuous classification of NTSs according to ICD-10 codes will enable us to characterise animal research with high precision and high resolution. Priorities and developments in research involving animals can be identified prospectively, and evidence-based arguments in favour of 3R research projects can be deduced. Our classification strategy has several benefits for different parties concerned with animal experiments: (1) For the public, a higher transparency regarding the benefits of current and upcoming in vivo research activities can be achieved. The information can contribute to informed opinions about animal experimentation, specifically in research areas involving controversial animal experiments. (2) Animal welfare can be improved, as detailed analyses of research areas with high numbers of in vivo projects as well as matching NTSs with respective authorised animal numbers could identify fields where 3R measures would be most efficient. Scientists and third-party funders looking for reasonable and innovative 3R research fields can utilise this information. (3) Researchers and funding bodies can benefit from the ample information provided in the NTSs, as future trends in in vivo biomedical research can be easily derived. Moreover, scientists applying for funding for ‘alternatives to animal experiments’ will be able to provide the precise number of animals that could be saved as a result of the planned project. Such numbers are often requested by funding institutions, such as the Wellcome Trust, National Centre for the Replacement, Refinement & Reduction of Animal in Research (NC3R), the German Centre for the Protection of Laboratory Animals (Bf3R), the German Federal Ministry of Education and Research (BMBF), the Organisation of the German Research Foundation (DFG), and Stiftung zur Forschung und Förderung von Ersatz- und Ergänzungsmethoden zur Einschränkung von Tierversuchen (The SET Foundation).

The ICD-10 classification of German NTSs will be further developed. The next steps involve the technical implementation of ICD-10 classes in *AnimalTestInfo* so that scientists can voluntarily allocate the most applicable ICD-10 code or target group to their project. In the future, NTSs shall be classified to ‘4-character subcategories’, which will allow for deeper data analyses. In addition to the ‘benefits’ field, we plan to evaluate statements mentioned in the fields ‘harms’ and ‘3R measures’. A box in which researchers can consistently indicate the expected degree of harm will be included. Here, we hope to gain information on implemented reduction and refinement measures within a specific research area. A detailed indication of the applied refinement measures would benefit other researchers working in the same field and could aid in improving the reproducibility of results.

To enable the targeted development of new 3R strategies, scientists could use the opportunity to provide more detailed information about their project, including the underlying hypothesis and study design. Finally, detailed information on animal experimentation and the resulting 3R research activities can contribute to replacing or reducing animal testing and to the implementation of refinement measures minimising pain, suffering, distress, or lasting harm in animals used for research.

## Materials and methods

### Data management and database construction

In Germany, NTSs are published in the web-based, searchable database *AnimalTestInfo* (https://animaltestinfo.de/), which was created and is maintained by the BfR [6]. All NTSs of authorised German scientific projects using animals are published, including projects classified as severe or authorised for the use of nonhuman primates. In Germany, projects that are subject to a simplified administrative procedure, e.g., testing necessary to satisfy regulatory requirements, do not require an NTS and, therefore, are not represented in the database. It is essential to mention that NTSs can involve experiments lasting up to 5 years; therefore, the number of NTSs for a particular year does not reflect the number of animals used annually and retrospectively reported by each Member State of the EU. The data requested in NTSs are based on the working document on nontechnical project summaries by the national competent authorities for the implementation of Directive 2010/63/EU on the protection of animals used for scientific purposes [14].

The relational database management system Informix was used to create and maintain a database with 16 tables. Four of the tables contain the information extracted directly from the NTSs, such as title, number and species of animals to be used, and objectives and potential benefits of the project. Another 4 tables were used to store indexing data, including ICD-10 codes, and the other tables contained catalogues and redundant data used to speed up searches. The application was written in CFML and JavaScript for the Adobe ColdFusion web application development platform.

In the principle workflow for submitting data to the database, researchers first log in to the database and create an NTS, which receives an individual ID. The entire procedure complies with the current Federal Data Protection Act. The researcher then sends the ID for its NTS together with the corresponding application form for the approval of an animal experiment to the competent authority. The competent authority ensures that the content of the NTS is accurate and corresponds with the project application. With the approval of the application, the NTS is activated in the database and made visible to the public.

### Dataset

For this pilot study, duly completed NTSs transferred to us in 2014 (*n* = 2,328) and 2015 (*n* = 2,970), i.e., a total of 5,298 NTSs, were analysed. A few NTSs (*n* = 54) were excluded from the analysis because the number of animals was not specified. For technical reasons, there is a 3-month time lag between the receipt and publication of an NTS in *AnimalTestInfo*. Hence, not all of the analysed NTSs were uploaded in the database in the years of receipt, i.e., some were published at the beginning of the following year (2015 and 2016, respectively).

The 5,298 NTS foresee the use of 7,872,775 animals (in 2014, *n* = 2,816,639; in 2015, *n* = 5,056,139). The number of animals per NTS varied greatly, with a range from 1 (cattle) to 394,560 animals (other fish).

Most NTSs indicate the use of fewer than 10,000 animals. Approximately 2% of all duly submitted NTS indicate the use of 10,000 or more animals, i.e., 29 NTSs in 2014 and 74 NTSs in 2015. This small proportion of NTSs (i.e., 103 in total) had a disproportionate influence on the results when NTSs were analysed according to animal numbers (see S3 Fig). Therefore, the 103 NTSs indicating the use of 3,352,066 animals in total were excluded from the general analysis and were analysed separately (see S1, S2 and S3 Figs and S1 Text). The threshold of 10,000 animals was chosen for pragmatic reasons. The use of other thresholds was not evaluated but is planned for future research projects.

### ICD-10 classification-based target group allocation

NTSs were classified by 3 indexers. The 2 main indexers were documentation assistants experienced in the area of terminology (use of thesauri, e.g., MeSH). The third indexer, an experienced scientist, was consulted by the 2 documentation assistants only if an NTS could not be easily attributed to an ICD-10 code or addressed target groups other than ‘patients’, ‘wild animals’, or ‘farm and domestic animals’ (an overview of the workflow is depicted in S5 Fig). Indexing was assisted by a standard operation procedure for classification (available in German in the OpenAgrar repository; https://doi.org/10.17590/20171025-154025) and online tools [11, 12].

Each NTS was classified by a single indexer. NTSs were opened individually and statements in the fields ‘title’ and ‘benefits’ were evaluated. First, NTSs were allocated to one of the following 5 target groups: ‘patients’, ‘pure basic research’, ‘consumers’, ‘farm and domestic animals’, ‘laboratory animals’, and ‘wild animals’. This supported classification-based information retrieval in cases in which ICD-10 codes were not applicable. The allocated target group refers to the population that is intended to benefit obviously from the results of the respective research project and was defined as follows: the target group ‘patients’ refers to NTSs in which a corresponding ICD-10 code could be allocated based on the statements given by applicants, i.e., when a target disease was mentioned. The target group ‘pure basic research’ was assigned if the planned research is intended to increase general biological knowledge and is not primarily application oriented. The target group ‘consumers’ was attributed to animal research considering the harmfulness or safety of consumer products, chemicals, or pesticides, including research in the areas of occupational safety and health. The target group ‘farm and domestic animals’ was attributed to veterinary research or experimental farm animal husbandry. The target group ‘laboratory animals’ included experimental laboratory animal science, e.g., the development of refinement measures. The target group ‘wild animals’ refers to, for example, research on conservation measures or testing for environmental toxicity. In cases in which NTS statements did not allow for an unequivocal assignment of a target group or in cases in which the planned animal usage was presented as an undesignated service for experimental in vivo studies (e.g., establishment of models or breeding), the label ‘no classification’ was used.

NTSs assigned to the target group ‘patient’ were further assigned an ICD-10-GM-2016 code. NTSs obtained in 2014 were classified to the ICD-10 chapter level only, except for those assigned to chapter II, *Neoplasms*, which were assigned to blocks of 3-character categories. The construction of an assisting IT-tool was completed in spring 2016; all NTSs obtained in 2015 were classified into a block of 3-character category with the help of this tool (but only chapters II, VI, and IX were further evaluated in this pilot study). NTSs were indexed according to the month of receipt and a list containing the allocated ICD-10 codes was exported for subsequent analyses implemented in MS Excel. The resulting MS Excel files can be retrieved from the OpenAgrar repository (https://doi.org/10.17590/20171025-153520).

A retrospective evaluation of the indexing consistency between the 2 main indexers was conducted. The 2 documentation assistants independently classified 100 randomly chosen (using an Informix random number generator) NTSs approved in 2015. The number and percentage of NTSs with matching ICD-10 codes were calculated. The analysis revealed a concordance of 95.5% for ICD-10 chapter and 80.6% for blocks of 3-character categories.

### Statistical analyses

Data of NTSs per target group (Fig 1), NTSs per ICD-10 chapter (Fig 2), and NTSs per block of 3-character category for chapters II, VI, and IX (Fig 3) are presented as percentages of the total NTS numbers. Please note that only in Fig 1 are all duly completed NTSs submitted in 2014 and 2015 depicted (*n* = 5,298). For data presented in Figs 2–4, NTSs referring to 10,000 animals or more (*n* = 103) were excluded from analyses. The separate analyses of these data are shown in the Supporting Information (S1–S3 Figs). All numerical values are mathematically rounded up or down to whole numbers.

For the comparison of NTSs allocated to the 9 ICD-10 blocks of 3-character categories (C15–C26, C69–C72, C81–C96, G10–G14, G30–G32, G35–G37, I30–I52, I60–I69, and I70–I79) shown in Fig 4, animal numbers per NTS were calculated and data are shown as box plots with medians [25th and 75th percentiles]. To facilitate comparisons, only those projects that planned to use mice alone for their research were included in this analysis. Statistical analyses were conducted using *R* [27]. Data were analysed by Kruskal–Wallis tests, followed by Wilcoxon rank sum tests. Differences in medians with *p* < 0.05 were considered statistically significant. Figs 1 and 4 and S1–S3 Figs were generated using SigmaPlot version 13 (Systat Software, Inc., San Jose, USA); Figs 2 and 3 and S4 Fig were generated using MS Excel (Microsoft Corp., Redmond, USA).

## Supporting information

S1 Fig Percentages of target groups for all animals. Distribution of the numbers of animals included in all NTSs obtained in 2014 (2,816,636 animals) and 2015 (5,056,139 animals) per target group. Data are presented as percentages of the total number of animals in NTSs of the respective years. Deviations from 100% are due to mathematical rounding. The majority of animals belonged to the target group ‘patients’ (67% or 1,883,116 animals in 2014 and 59% or 2,971,632 animals in 2015). A large proportion of animals of corresponding NTSs could not be assigned to a specific target group and thus were allocated to the group ‘no classification’, including 23%, i.e., 657,026 animals, in 2014 and 16%, i.e., 788,336 animals, in 2015. The other corresponding animal numbers were distributed over the groups ‘pure basic research’ (6% in 2014 and 12% in 2015), ‘consumers’ (1% in 2014 and <1% in 2015), ‘farm and domestic animals’ (1% each), ‘laboratory animals’ (<1% each), and ‘wild animals’ (2% in 2014 and 12% in 2015). See also S1 Data and Fig 1 for the corresponding NTS allocations. NTS, nontechnical summary.
(TIF)

S2 Fig Percentages of target groups for animals of NTSs with 10,000 animals or more. Distribution of animals across target groups for those 103 NTSs (29 NTSs in 2014 and 74 NTSs in 2015) that indicated the use of 10,000 or more animals. This analysis includes 880,918 animals in 2014 (corresponding to approximately 30% of the total animal number) and 2,471,148 animals in 2015 (corresponding to approximately 50% of the total animal number). Data are presented as percentages of the total number of animals reported in the respective years. Deviations from 100% are due to mathematical rounding. The proportion of animals in the target group ‘patients’ was very similar in 2014 (32%) and 2015 (36%). ‘No classification’ constituted the largest group in 2014, with 57%, and the second largest group in 2015, with 27%. The target group ‘pure basic research’ accounted for 9% of animals in 2014 and for 16% of animals in 2015. For 2014, only 2% of animals were assigned to the target group ‘wild animals’, whereas in 2015, a large proportion, i.e., 21% of approved animals, was allocated to this target group. This high value is mainly attributed to a single NTS indicating the use of 394,560 fish. The remaining target groups were not represented. See also S1 Data. NTS, nontechnical summary.
(TIF)

S3 Fig Percentages of target groups for animals of NTSs indicating fewer than 10,000 animals. Distribution of numbers of animals included in NTSs obtained in 2014 (1,935,718 animals) and 2015 (2,584,991 animals) in target groups related to envisaged research. Note that data from 29 (2014) and 74 (2015) NTSs that foresee animal numbers of greater than or equal to 10,000 in a single application were excluded from this analysis (*n* = 880,918 animals in 2014; *n* = 2,471,148 animals in 2015). Data are presented as percentages of the total number of animals approved for use in the respective years. Deviations from 100% are due to rounding. Using this dataset, 82% (2014) and 80% (2015) of animals were related to research assigned to the target group ‘patients’; 8% and 5% of animals for NTSs indicating fewer than 10,000 animals were assigned to ‘no classification’ in 2014 and 2015, respectively. The target group ‘pure basic research’ accounted for 5% of animals in 2014 and for 9% of animals in 2015. For 2014, we classified 2% of animals in the target group ‘wild animals’ and 3% in 2015. The other corresponding animal numbers were distributed over the groups ‘consumers’ (2% in 2014 and 1% in 2015), ‘farm and domestic animals’ (1% and 2%, respectively), and ‘laboratory animals’ (<1% each). See also S1 Data. NTS, nontechnical summary.
(TIF)

S4 Fig Animal numbers per ICD-10 chapter. Shown are animal numbers for NTSs of the target group ‘patients’ allocated to 22 ICD-10 chapters (see Fig 2), i.e., 1,595,309 animals in 2014 (inner ring) and 2,073,656 animals in 2015 (outer ring) indicated in NTSs (abbreviated titles of categories and percentages are indicated in brackets). NTSs with 10,000 animals or more were excluded; chapter labels are abridgements. Data are presented as percentages of the total number of animals of the target group ‘patients’. Deviations from 100% are due to mathematical rounding. Additionally, 12% and 8% of authorised animals in 2014 and 2015, respectively, were labelled ‘multiple chapters’, as they could be allocated to more than one ICD-10 chapter. Note that animal numbers provided in NTSs can cover experiments lasting up to 5 years. Hence, these figures cannot be compared with the official annual statistical reports about the respective animals. See also S1 Data and Fig 2 for the corresponding NTS allocations. ICD, International Classification of Diseases and Related Health Problems; NTS, nontechnical summary.
(TIF)

S5 Fig Workflow of target group allocation and ICD-10 classification. This is an overview of the workflow for target group allocation and ICD-10 classification. The workflow is described in more detail in the Materials and methods. A standard operation procedure for classification is available in German in the OpenAgrar repository (https://doi.org/10.17590/20171025-154025). ICD, International Classification of Diseases and Related Health Problems.
(TIF)

S1 Text Supporting discussion of S1–S4 Figs. Here, the relationship between NTSs and corresponding animal numbers are discussed. NTS, nontechnical summary.
(DOCX)

S2 Text Consideration of 3 classification systems. The advantages and disadvantages of 3 classification systems, i.e., the MeSH thesaurus, the EU classification according to the Implementing Decision 2012/707/EU, and the ICD-10, are discussed. ICD, International Classification of Diseases and Related Health Problems; MeSH, Medical Subject Headings.
(DOCX)

S1 Data Datasets to generate Figs 1, 2, 3 and 4 as well as S1, S2, S3 and S4 Figs. (XLSX)

S1 Table List of ICD-10 chapters and blocks of 3-character categories. This table lists the ICD-10 chapters and blocks of 3-character categories according to WHO. ICD, International Classification of Diseases and Related Health Problems; WHO, World Health Organization.
(DOCX)

## Abbreviations

3R: replace, reduce, and refine
Bf3R: German Centre for the Protection of Laboratory Animals
BfR: German Federal Institute for Risk Assessment
BMBF: German Federal Ministry of Education and Research
DFG: Organisation of the German Research Foundation
EU: European Union
ICD: International Classification of Diseases and Related Health Problems
NC3R: National Centre for the Replacement, Refinement & Reduction of Animal in Research
NTS: nontechnical summary
The SET Foundation: Stiftung zur Forschung und Förderung von Ersatz- und Ergänzungsmethoden zur Einschränkung von Tierversuchen
WHO: World Health Organization
