# Supplementary figures and images for: Rethinking 3R strategies: Digging deeper into AnimalTestInfo promotes transparency in in vivo biomedical research
Source: PLoS Biol. 2017 Dec 14;15(12):e2003217. doi: 10.1371/journal.pbio.2003217 (PMC5730105; doi:10.1371/journal.pbio.2003217)

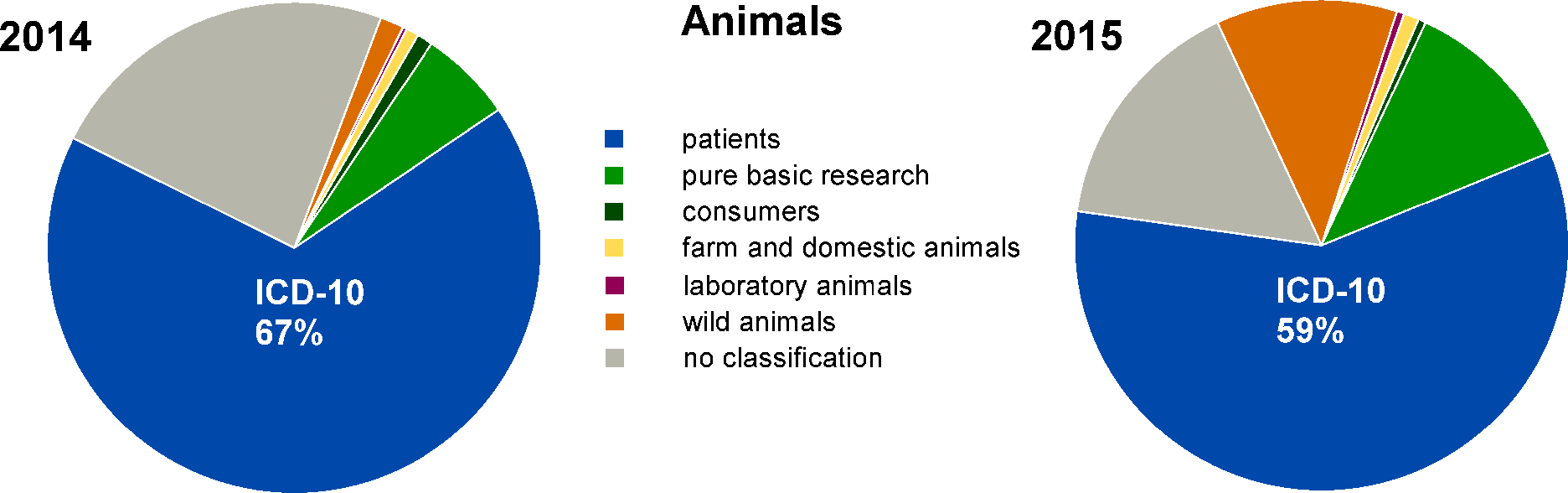

Supplement: S1 Fig — Distribution of the numbers of animals included in all NTSs obtained in 2014 (2,816,636 animals) and 2015 (5,056,139 animals) per target group. Data are presented as percentages of the total number of animals in NTSs of the respective years. Deviations from 100% are due to mathematical rounding. The majority of animals belonged to the target group ‘patients’ (67% or 1,883,116 animals in 2014 and 59% or 2,971,632 animals in 2015). A large proportion of animals of corresponding NTSs could not be assigned to a specific target group and thus were allocated to the group ‘no classification’, including 23%, i.e., 657,026 animals, in 2014 and 16%, i.e., 788,336 animals, in 2015. The other corresponding animal numbers were distributed over the groups ‘pure basic research’ (6% in 2014 and 12% in 2015), ‘consumers’ (1% in 2014 and <1% in 2015), ‘farm and domestic animals’ (1% each), ‘laboratory animals’ (<1% each), and ‘wild animals’ (2% in 2014 and 12% in 2015). See also S1 Data and Fig 1 for the corresponding NTS allocations. NTS, nontechnical summary. (TIF) [file pbio.2003217.s001.tif]

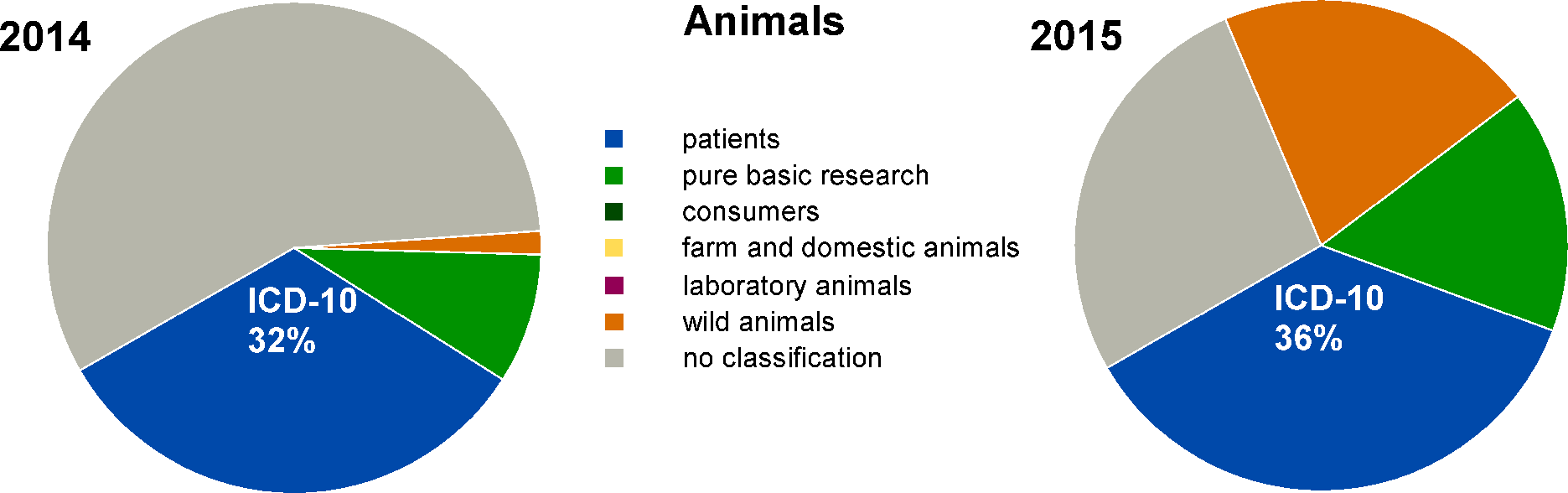

Supplement: S2 Fig — Distribution of animals across target groups for those 103 NTSs (29 NTSs in 2014 and 74 NTSs in 2015) that indicated the use of 10,000 or more animals. This analysis includes 880,918 animals in 2014 (corresponding to approximately 30% of the total animal number) and 2,471,148 animals in 2015 (corresponding to approximately 50% of the total animal number). Data are presented as percentages of the total number of animals reported in the respective years. Deviations from 100% are due to mathematical rounding. The proportion of animals in the target group ‘patients’ was very similar in 2014 (32%) and 2015 (36%). ‘No classification’ constituted the largest group in 2014, with 57%, and the second largest group in 2015, with 27%. The target group ‘pure basic research’ accounted for 9% of animals in 2014 and for 16% of animals in 2015. For 2014, only 2% of animals were assigned to the target group ‘wild animals’, whereas in 2015, a large proportion, i.e., 21% of approved animals, was allocated to this target group. This high value is mainly attributed to a single NTS indicating the use of 394,560 fish. The remaining target groups were not represented. See also S1 Data. NTS, nontechnical summary. (TIF) [file pbio.2003217.s002.tif]

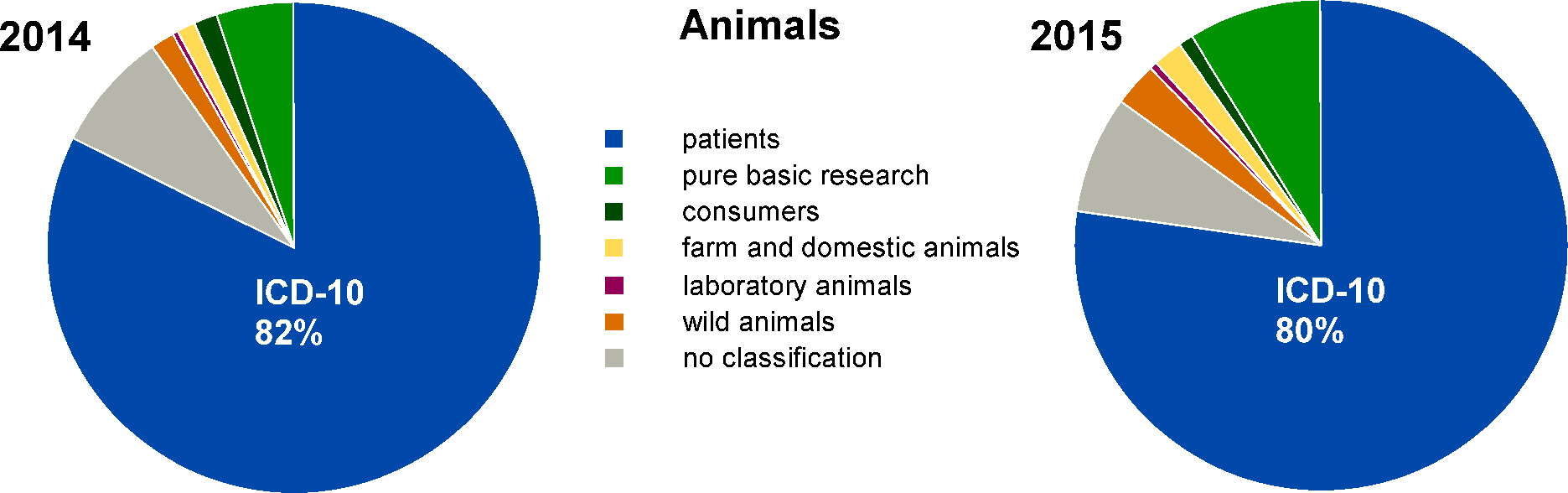

Supplement: S3 Fig — Distribution of numbers of animals included in NTSs obtained in 2014 (1,935,718 animals) and 2015 (2,584,991 animals) in target groups related to envisaged research. Note that data from 29 (2014) and 74 (2015) NTSs that foresee animal numbers of greater than or equal to 10,000 in a single application were excluded from this analysis (n = 880,918 animals in 2014; n = 2,471,148 animals in 2015). Data are presented as percentages of the total number of animals approved for use in the respective years. Deviations from 100% are due to rounding. Using this dataset, 82% (2014) and 80% (2015) of animals were related to research assigned to the target group ‘patients’; 8% and 5% of animals for NTSs indicating fewer than 10,000 animals were assigned to ‘no classification’ in 2014 and 2015, respectively. The target group ‘pure basic research’ accounted for 5% of animals in 2014 and for 9% of animals in 2015. For 2014, we classified 2% of animals in the target group ‘wild animals’ and 3% in 2015. The other corresponding animal numbers were distributed over the groups ‘consumers’ (2% in 2014 and 1% in 2015), ‘farm and domestic animals’ (1% and 2%, respectively), and ‘laboratory animals’ (<1% each). See also S1 Data. NTS, nontechnical summary. (TIF) [file pbio.2003217.s003.tif]

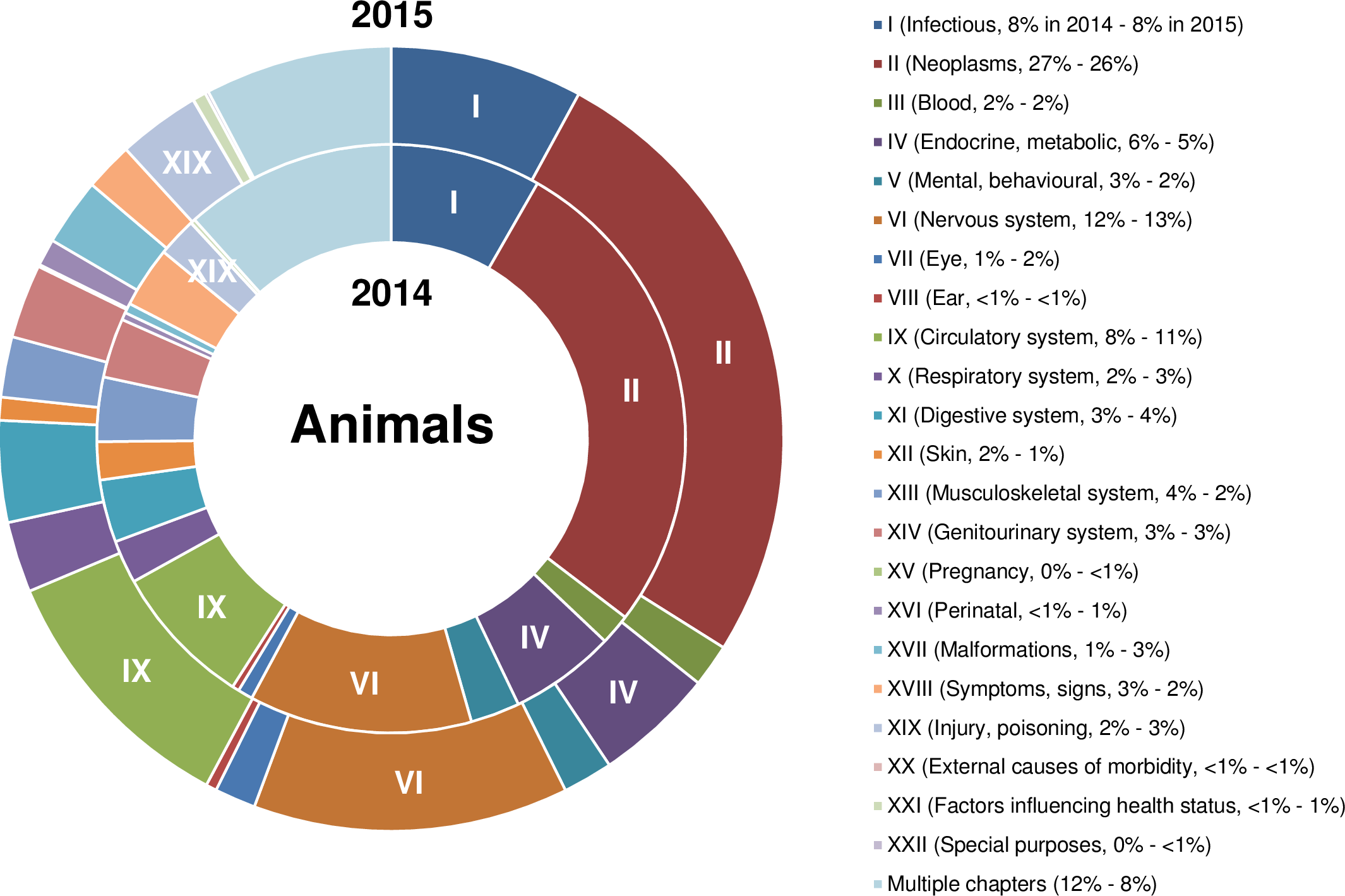

Supplement: S4 Fig — Shown are animal numbers for NTSs of the target group ‘patients’ allocated to 22 ICD-10 chapters (see Fig 2), i.e., 1,595,309 animals in 2014 (inner ring) and 2,073,656 animals in 2015 (outer ring) indicated in NTSs (abbreviated titles of categories and percentages are indicated in brackets). NTSs with 10,000 animals or more were excluded; chapter labels are abridgements. Data are presented as percentages of the total number of animals of the target group ‘patients’. Deviations from 100% are due to mathematical rounding. Additionally, 12% and 8% of authorised animals in 2014 and 2015, respectively, were labelled ‘multiple chapters’, as they could be allocated to more than one ICD-10 chapter. Note that animal numbers provided in NTSs can cover experiments lasting up to 5 years. Hence, these figures cannot be compared with the official annual statistical reports about the respective animals. See also S1 Data and Fig 2 for the corresponding NTS allocations. ICD, International Classification of Diseases and Related Health Problems; NTS, nontechnical summary. (TIF) [file pbio.2003217.s004.tif]

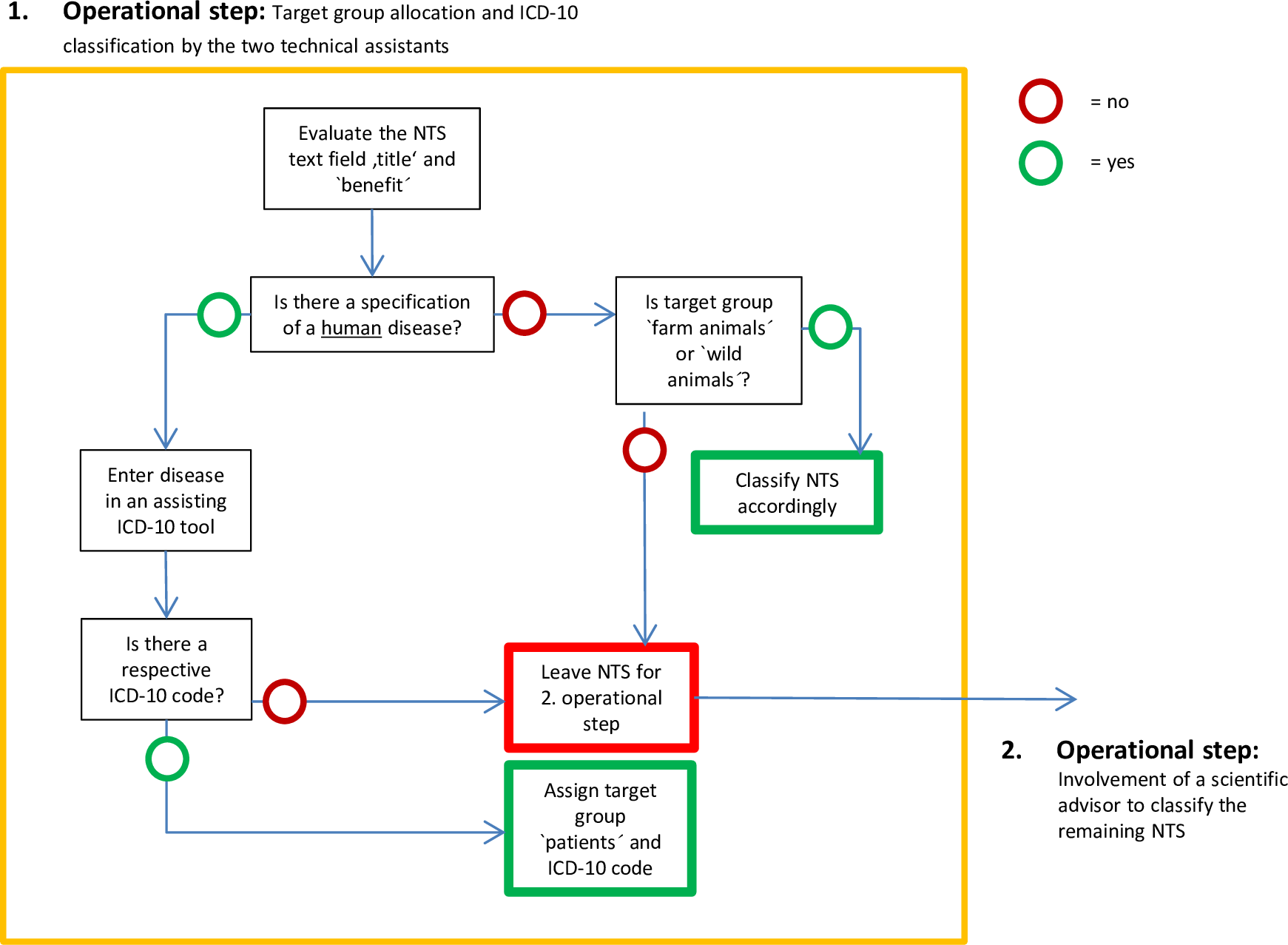

Supplement: S5 Fig — This is an overview of the workflow for target group allocation and ICD-10 classification. The workflow is described in more detail in the Materials and methods. A standard operation procedure for classification is available in German in the OpenAgrar repository (https://doi.org/10.17590/20171025-154025). ICD, International Classification of Diseases and Related Health Problems. (TIF) [file pbio.2003217.s005.tif]
