# Supplementary material for: Rethinking 3R strategies: Digging deeper into AnimalTestInfo promotes transparency in in vivo biomedical research
Source: PLoS Biol. 2017 Dec 14;15(12):e2003217. doi: 10.1371/journal.pbio.2003217 (PMC5730105; doi:10.1371/journal.pbio.2003217)
Supplement: S1 Text — Here, the relationship between NTSs and corresponding animal numbers are discussed. NTS, nontechnical summary. (DOCX) [file pbio.2003217.s006.docx]

**Supporting discussion of S1–S4 Figs**

**Analyses of animal numbers**

When analysing the animal numbers mentioned in the NTSs, as summarised in Fig 1, we noticed a discrepancy in the target group distribution between NTS numbers (Fig 1) and the corresponding animal numbers (S1 Fig): Although the majority of animals were found in the target group ‘patients’, the percentage was much lower (67% or 1,883,116 animals in 2014 and 59% or 2,971,632 animals in 2015) than expected from the results shown in Fig 1 (81% of NTSs in 2014 and 80% in 2015). In addition, a substantially larger proportion of animals than of corresponding NTSs could not be assigned to a specific target group and thus were allocated to the group ‘no classification’, i.e., 23% or 657,026 animals in 2014 and 16% or 788,336 animals in 2015 compared to 6% of NTSs in 2014 and 4% in 2015. The analysis further revealed great variation in the distribution of animal numbers across categories among the 2 consecutive years for some of the other target groups, including ‘pure basic research’ (6% in 2014 and 12% in 2015), ‘consumers’ (1% in 2014 and <1% in 2015), ‘farm and domestic animals’ (1% each), ‘laboratory animals’ (<1% each), and ‘wild animals’ (2% in 2014 and 12% in 2015), whereas NTSs were essentially equally distributed in 2014 and 2015. One would expect the target group distribution to be similar, irrespective of whether NTS numbers or animal numbers are analysed. However, this would only be true if each NTS contains comparable animal numbers. Thus, we assumed that this discrepancy was caused by single NTSs including large numbers of animals.

To account for this issue, we analysed NTSs indicating 10,000 animals per NTS or more separately. The limit of 10,000 animals was a pragmatic approach as we noticed that these NTSs account for 1% of NTSs in 2014 (i.e., 29 NTSs) and 2.5% in 2015 (i.e., 74 NTSs), but represent a large proportion of animals, i.e., 880,918 animals in 2014 (corresponds to approximately 30% of the total animal number) and 2,471,148 animals in 2015 (corresponds to approximately 50% of the total animal number). Even at first glance, the target group distribution shown in S2 Fig differs strikingly from that shown in Fig 1 and S1 Fig and differs substantially between the 2 years. However, when analysing animal numbers for NTSs referring to fewer than 10,000 animals (see S3 Fig), it becomes apparent that the numbers are in good accordance with NTS numbers in Fig 1 and only minor deviations in the range of 1%–2% were detected.

S1–S3 Figs show that the 103 NTSs reporting 10,000 or more animals would have had substantial and disproportionate impacts when analysing animal numbers. It is important to note that these few NTSs influence assessments of animal numbers per target group or per ICD-10 code, but not the analysis of pure NTS numbers. A comparison of the results obtained for 2 consecutive years argued for the exclusion of NTSs with equal to or greater than 10,000 animals. Data obtained in 2014 and 2015 were comparable when excluding NTSs reporting high numbers of animals (see S3 Fig), but we observed high variation between years when data for NTSs involving 10,000 animals or more were included in the analysis (see S1 and S2 Fig). S1 Fig also reveals that NTSs assigned to ‘no classification’ comprised large numbers of animals, i.e. 657,026 animals in 2014 and 788,336 animals in 2015. This emphasizes the need for providing feedback to applicants regarding the quality of their statements, and to expand the currently scheduled ‘target groups’.

**Analysis of animal numbers with respect to ICD-10 classification**

S4 Fig gives an overview of the animal numbers for NTSs shown in Fig 2 according to ICD-10 chapters. Data shown in S4 Fig and Fig 2 exclude animals from the 103 NTSs reporting 10,000 animals or more and, thus, include 1,595,309 animals in 2014 and 2,073,656 animals in 2015. Of note, the animal numbers reported in NTSs can cover experiments lasting up to 5 years. Therefore, these figures cannot be compared with the official annual statistical reports. The distribution of animal numbers over the ICD-10 chapters seems to roughly reflect the distribution of NTSs over the ICD-10 chapters, as shown in Fig 2. However, on closer examination, for some research fields, e.g., *Neoplasms* (chapter II) and *Diseases of the nervous system* (chapter VI), a higher number of animals per NTS seemed to be applied when compared to animal numbers for other research areas, e.g. *Diseases of the circulatory system* (chapter IX). This observation stimulated us to further analyse this aspect. As an example, we analysed the distribution of animal numbers per NTS from 9 different blocks of 3-character categories of 3 ICD-10 chapters (see Fig 4). As animal species could be a confounding factor for the discrepancies between the proportions of NTSs numbers and animal numbers, we only analysed those NTSs involving mice.
