# Supplementary material for: Rethinking 3R strategies: Digging deeper into AnimalTestInfo promotes transparency in in vivo biomedical research
Source: PLoS Biol. 2017 Dec 14;15(12):e2003217. doi: 10.1371/journal.pbio.2003217 (PMC5730105; doi:10.1371/journal.pbio.2003217)
Supplement: S2 Text — The advantages and disadvantages of 3 classification systems, i.e., the MeSH thesaurus, the EU classification according to the Implementing Decision 2012/707/EU, and the ICD-10, are discussed. ICD, International Classification of Diseases and Related Health Problems; MeSH, Medical Subject Headings. (DOCX) [file pbio.2003217.s007.docx]

**Consideration of 3 classification systems for indexing nontechnical project summaries**

Three established classification systems were considered for indexing statements provided in the ‘benefits’ and ‘title’ sections of NTSs: (1) the Medical Subject Headings (MeSH) thesaurus, (2) the EU classification according to the Implementing Decision 2012/707/EU, and (3) the ICD-10.

1. The MeSH thesaurus is a controlled vocabulary produced by the National Library of Medicine and is used for indexing, cataloguing, and searching for biomedical and health-related information and documents in PubMed/Medline (https://www.nlm.nih.gov/mesh/introduction.html). Each MeSH term (‘descriptor’) is defined by a scope note that specifies the meaning of the term. The MeSH thesaurus is organised as a hierarchical tree, from most general to most specific terms. Individual terms may be assigned to one or several branches of the tree. Thus, the term ‘breast cancer’ and its MeSH-descriptor ‘breast neoplasms’ are grouped under the umbrella term ‘neoplasms’, but also under the umbrella term ‘skin diseases’. NTSs describing planned experimental research into ‘breast cancer’ therefore would have been assigned to ‘MeSH: neoplasms’, but also ‘MeSH: skin diseases’. While this duplication does not pose a problem for high recall-adjusted searches, it is a problem for quantitative analysis. One single NTS may be counted several times in the above example, once as constituent of ‘experimental research into neoplasms’, once as constituent of ‘experimental research into skin diseases’. In addition to some ambiguous scope notes, this major disadvantage moved us to not use the MeSH-thesaurus as the chief classification system for *AnimalTestInfo*.
2. The EU classification (2012/707/EU, ANNEX II, part A) was set up to provide a common format for reporting the statistical information required under Article 54 (2) of the Directive 2010/63/EU [1, 2]. It is organised as a flowchart starting with ‘type of animal’, proceeding to ‘purposes’ (e.g. basic research, translational and applied research, regulatory use, and routine production), to subcategories of theses purposes (e.g. ‘basic research studies: oncology’ and ‘translational and applied research: human cancer’). Only the subcategories under ‘regulatory use and routine production’ allow for a more specific assignment. Other subcategories provide just one hierarchical level. There are some scope notes attached in part B of ANNEX II; however, these scope notes leave too much room for individual interpretation. We considered the EU classification too simplistic to be utilised as the classification system for the NTSs deposited in *AnimalTestInfo*.
3. The ‘International Classification of Diseases and Related Health Problems’ (ICD) is the international standard for defining and reporting diseases and health conditions, and is used to monitor death and disease rates [3]. Diseases, disorders, injuries, and other related health conditions are organised in 22 chapters. The ICD codes provide several hierarchical layers: The 22 chapters are divided into ‘blocks of 3-character categories’, ‘3-character categories’, and ‘4-character subcategories’ (see also Table 1). Thus, the ICD facilitates a high resolution of biomedical research, with high precision. There are numerous tools for the assignment of ICD-10 codes available [4-6]. The ICD classification is subject to continuous revision; the 11th version will presumably be finalised in 2018. Some countries, including Germany, have adopted the ICD for clinical use.

As the analysed NTSs are published in German, we used the German modification of the ICD-10, Version 2016 (ICD-10-GM-2016).

**References**

1. Directive 2010/63/EU of the European Parliament and of the Council of 22 September 2010 on the protection of animals used for scientific purposes. 22 September 2010. Available from: http://eur-lex.europa.eu/LexUriServ/LexUriServ.do?uri=OJ:L:2010:276:0033:0079:en:PDF. Cited 23 October 2017.

2. 2012/707/EU: Commission Implementing Decision of 14 November 2012 establishing a common format for the submission of the information pursuant to Directive 2010/63/EU of the European Parliament and of the Council on the protection of animals used for scientific purposes. 14 November 2012. Available from: http://eur-lex.europa.eu/legal-content/EN/TXT/PDF/?uri=CELEX:32012D0707&from=EN. Cited 23 October 2017.

3. World Health Organisation (WHO). Classification of diseases. [Cited 23 October 2017]. Available from: http://www.who.int/classifications/icd/en/.

4. Krollner B, Krollner DM. ICD-code. [Cited 23 October 2017]. Available from: <http://www.icd-code.de/>.

5. Liebermann B. ICD Scout. [Cited 23 October 2017]. Available from: <http://www.icdscout.de/>.

6. World Health Organisation. ICD-10 Interactive Self Learning Tool. Accessed September 21st 2017. Available from: <http://apps.who.int/classifications/apps/icd/icd10training/>.
