# Supplementary material for: Rethinking 3R strategies: Digging deeper into AnimalTestInfo promotes transparency in in vivo biomedical research
Source: PLoS Biol. 2017 Dec 14;15(12):e2003217. doi: 10.1371/journal.pbio.2003217 (PMC5730105; doi:10.1371/journal.pbio.2003217)
Supplement: S1 Table — This table lists the ICD-10 chapters and blocks of 3-character categories according to WHO. ICD, International Classification of Diseases and Related Health Problems; WHO, World Health Organization. (DOCX) [file pbio.2003217.s009.docx]

**S1 Table. List of ICD-10 chapters and blocks of 3-character categories.**

| **ICD-10 chapters** | **ICD-10 subcategories** |
| --- | --- |
| **I** Certain infectious and parasitic diseases | A00-A09 Intestinal infectious diseases  A15-A19 Tuberculosis  A20-A28 Certain zoonotic bacterial diseases  A30-A49 Other bacterial diseases  A50-A64 Infections with a predominantly sexual mode of transmission  A65-A69 Other spirochaetal diseases  A70-A74 Other diseases caused by chlamydiae  A75-A79 Rickettsioses  A80-A89 Viral infections of the central nervous system  A92-A99 Arthropod-borne viral fevers and viral haemorrhagic fevers  B00-B09 Viral infections characterized by skin and mucous membrane lesions  B15-B19 Viral hepatitis  B20-B24 Human immunodeficiency virus [HIV] disease  B25-B34 Other viral diseases  B35-B49 Mycoses  B50-B64 Protozoal diseases  B65-B83 Helminthiases  B85-B89 Pediculosis, acariasis and other infestations  B90-B94 Sequelae of infectious and parasitic diseases  B95-B98 Bacterial, viral and other infectious agents  B99-B99 Other infectious diseases |
| **II** Neoplasms | C00-C14 Malignant neoplasms of lip, oral cavity and pharynx  C15-C26 Malignant neoplasms of digestive organs  C30-C39 Malignant neoplasms of respiratory and intrathoracic organs  C40-C41 Malignant neoplasms of bone and articular cartilage  C43-C44 Melanoma and other malignant neoplasms of skin  C45-C49 Malignant neoplasms of mesothelial and soft tissue  C50-C50 Malignant neoplasm of breast  C51-C58 Malignant neoplasms of female genital organs  C60-C63 Malignant neoplasms of male genital organs  C64-C68 Malignant neoplasms of urinary tract  C69-C72 Malignant neoplasms of eye, brain and other parts of central nervous system  C73-C75 Malignant neoplasms of thyroid and other endocrine glands  C76-C80 Malignant neoplasms of ill-defined, secondary and unspecified sites  C81-C96 Malignant neoplasms, stated or presumed to be primary, of lymphoid, haematopoietic and related tissue  C97-C97 Malignant neoplasms of independent (primary) multiple sites  D00-D09 In situ neoplasms  D10-D36 Benign neoplasms  D37-D48 Neoplasms of uncertain or unknown behaviour |
| **III** Diseases of the blood and blood-forming organs and certain disorders involving the immune mechanism | D50-D53 Nutritional anaemias  D55-D59 Haemolytic anaemias  D60-D64 Aplastic and other anaemias  D65-D69 Coagulation defects, purpura and other haemorrhagic conditions  D70-D77 Other diseases of blood and blood-forming organs  D80-D89 Certain disorders involving the immune mechanism |
| **IV** Endocrine, nutritional and metabolic diseases | E00-E07 Disorders of thyroid gland  E10-E14 Diabetes mellitus  E15-E16 Other disorders of glucose regulation and pancreatic internal secretion  E20-E35 Disorders of other endocrine glands  E40-E46 Malnutrition  E50-E64 Other nutritional deficiencies  E65-E68 Obesity and other hyperalimentation  E70-E90 Metabolic disorders |
| **V** Mental and behavioural disorders | F00-F09 Organic, including symptomatic, mental disorders  F10-F19 Mental and behavioural disorders due to psychoactive substance use  F20-F29 Schizophrenia, schizotypal and delusional disorders  F30-F39 Mood [affective] disorders  F40-F48 Neurotic, stress-related and somatoform disorders  F50-F59 Behavioural syndromes associated with physiological disturbances and physical factors  F60-F69 Disorders of adult personality and behaviour  F70-F79 Mental retardation  F80-F89 Disorders of psychological development  F90-F98 Behavioural and emotional disorders with onset usually occurring in childhood and adolescence  F99-F99 Unspecified mental disorder |
| **VI** Diseases of the nervous system | G00-G09 Inflammatory diseases of the central nervous system  G10-G14 Systemic atrophies primarily affecting the central nervous system  G20-G26 Extrapyramidal and movement disorders  G30-G32 Other degenerative diseases of the nervous system  G35-G37 Demyelinating diseases of the central nervous system  G40-G47 Episodic and paroxysmal disorders  G50-G59 Nerve, nerve root and plexus disorders  G60-G64 Polyneuropathies and other disorders of the peripheral nervous system  G70-G73 Diseases of myoneural junction and muscle  G80-G83 Cerebral palsy and other paralytic syndromes  G90-G99 Other disorders of the nervous system |
| **VII** Diseases of the eye and adnexa | H00-H06 Disorders of eyelid, lacrimal system and orbit  H10-H13 Disorders of conjunctiva  H15-H22 Disorders of sclera, cornea, iris and ciliary body  H25-H28 Disorders of lens  H30-H36 Disorders of choroid and retina  H40-H42 Glaucoma  H43-H45 Disorders of vitreous body and globe  H46-H48 Disorders of optic nerve and visual pathways  H49-H52 Disorders of ocular muscles, binocular movement, accommodation and refraction  H53-H54 Visual disturbances and blindness  H55-H59 Other disorders of eye and adnexa |
| **VIII** Diseases of the ear and mastoid process | H60-H62 Diseases of external ear  H65-H75 Diseases of middle ear and mastoid  H80-H83 Diseases of inner ear  H90-H95 Other disorders of ear |
| **IX** Diseases of the circulatory system | I00-I02 Acute rheumatic fever  I05-I09 Chronic rheumatic heart diseases  I10-I15 Hypertensive diseases  I20-I25 Ischaemic heart diseases  I26-I28 Pulmonary heart disease and diseases of pulmonary circulation  I30-I52 Other forms of heart disease  I60-I69 Cerebrovascular diseases  I70-I79 Diseases of arteries, arterioles and capillaries  I80-I89 Diseases of veins, lymphatic vessels and lymph nodes, not elsewhere classified  I95-I99 Other and unspecified disorders of the circulatory system |
| **X** Diseases of the respiratory system | J00-J06 Acute upper respiratory infections  J09-J18 Influenza and pneumonia  J20-J22 Other acute lower respiratory infections  J30-J39 Other diseases of upper respiratory tract  J40-J47 Chronic lower respiratory diseases  J60-J70 Lung diseases due to external agents  J80-J84 Other respiratory diseases principally affecting the interstitium  J85-J86 Suppurative and necrotic conditions of lower respiratory tract  J90-J94 Other diseases of pleura  J95-J99 Other diseases of the respiratory system |
| **XI** Diseases of the digestive system | K00-K14 Diseases of oral cavity, salivary glands and jaws  K20-K31 Diseases of oesophagus, stomach and duodenum  K35-K38 Diseases of appendix  K40-K46 Hernia  K50-K52 Noninfective enteritis and colitis  K55-K64 Other diseases of intestines  K65-K67 Diseases of peritoneum  K70-K77 Diseases of liver  K80-K87 Disorders of gallbladder, biliary tract and pancreas  K90-K93 Other diseases of the digestive system |
| **XII** Diseases of the skin and subcutaneous tissue | L00-L08 Infections of the skin and subcutaneous tissue  L10-L14 Bullous disorders  L20-L30 Dermatitis and eczema  L40-L45 Papulosquamous disorders  L50-L54 Urticaria and erythema  L55-L59 Radiation-related disorders of the skin and subcutaneous tissue  L60-L75 Disorders of skin appendages  L80-L99 Other disorders of the skin and subcutaneous tissue |
| **XIII** Diseases of the musculoskeletal system and connective tissue | M00-M25 Arthropathies  M30-M36 Systemic connective tissue disorders  M40-M54 Dorsopathies  M60-M79 Soft tissue disorders  M80-M94 Osteopathies and chondropathies  M95-M99 Other disorders of the musculoskeletal system and connective tissue |
| **XIV** Diseases of the genitourinary system | N00-N08 Glomerular diseases  N10-N16 Renal tubulo-interstitial diseases  N17-N19 Renal failure  N20-N23 Urolithiasis  N25-N29 Other disorders of kidney and ureter  N30-N39 Other diseases of urinary system  N40-N51 Diseases of male genital organs  N60-N64 Disorders of breast  N70-N77 Inflammatory diseases of female pelvic organs  N80-N98 Noninflammatory disorders of female genital tract  N99-N99 Other disorders of the genitourinary system |
| **XV** Pregnancy, childbirth and the puerperium | O00-O08 Pregnancy with abortive outcome  O10-O16 Oedema, proteinuria and hypertensive disorders in pregnancy, childbirth and the puerperium  O20-O29 Other maternal disorders predominantly related to pregnancy  O30-O48 Maternal care related to the fetus and amniotic cavity and possible delivery problems  O60-O75 Complications of labour and delivery  O80-O84 Delivery  O85-O92 Complications predominantly related to the puerperium  O94-O99 Other obstetric conditions, not elsewhere classified |
| **XVI** Certain conditions originating in the perinatal period | P00-P04 Fetus and newborn affected by maternal factors and by complications of pregnancy, labour and delivery  P05-P08 Disorders related to length of gestation and fetal growth  P10-P15 Birth trauma  P20-P29 Respiratory and cardiovascular disorders specific to the perinatal period  P35-P39 Infections specific to the perinatal period  P50-P61 Haemorrhagic and haematological disorders of fetus and newborn  P70-P74 Transitory endocrine and metabolic disorders specific to fetus and newborn  P75-P78 Digestive system disorders of fetus and newborn  P80-P83 Conditions involving the integument and temperature regulation of fetus and newborn  P90-P96 Other disorders originating in the perinatal period |
| **XVII** Congenital malformations, deformations and chromosomal abnormalities | Q00-Q07 Congenital malformations of the nervous system  Q10-Q18 Congenital malformations of eye, ear, face and neck  Q20-Q28 Congenital malformations of the circulatory system  Q30-Q34 Congenital malformations of the respiratory system  Q35-Q37 Cleft lip and cleft palate  Q38-Q45 Other congenital malformations of the digestive system  Q50-Q56 Congenital malformations of genital organs  Q60-Q64 Congenital malformations of the urinary system  Q65-Q79 Congenital malformations and deformations of the musculoskeletal system  Q80-Q89 Other congenital malformations  Q90-Q99 Chromosomal abnormalities, not elsewhere classified |
| **XVIII** Symptoms, signs and abnormal clinical and laboratory findings, not elsewhere classified | R00-R09 Symptoms and signs involving the circulatory and respiratory systems  R10-R19 Symptoms and signs involving the digestive system and abdomen  R20-R23 Symptoms and signs involving the skin and subcutaneous tissue  R25-R29 Symptoms and signs involving the nervous and musculoskeletal systems  R30-R39 Symptoms and signs involving the urinary system  R40-R46 Symptoms and signs involving cognition, perception, emotional state and behaviour  R47-R49 Symptoms and signs involving speech and voice  R50-R69 General symptoms and signs  R70-R79 Abnormal findings on examination of blood, without diagnosis  R80-R82 Abnormal findings on examination of urine, without diagnosis  R83-R89 Abnormal findings on examination of other body fluids, substances and tissues, without diagnosis  R90-R94 Abnormal findings on diagnostic imaging and in function studies, without diagnosis  R95-R99 Ill-defined and unknown causes of mortality |
| **XIX** Injury, poisoning and certain other consequences of external causes | S00-S09 Injuries to the head  S10-S19 Injuries to the neck  S20-S29 Injuries to the thorax  S30-S39 Injuries to the abdomen, lower back, lumbar spine and pelvis  S40-S49 Injuries to the shoulder and upper arm  S50-S59 Injuries to the elbow and forearm  S60-S69 Injuries to the wrist and hand  S70-S79 Injuries to the hip and thigh  S80-S89 Injuries to the knee and lower leg  S90-S99 Injuries to the ankle and foot  T00-T07 Injuries involving multiple body regions  T08-T14 Injuries to unspecified part of trunk, limb or body region  T15-T19 Effects of foreign body entering through natural orifice  T20-T32 Burns and corrosions  T33-T35 Frostbite  T36-T50 Poisoning by drugs, medicaments and biological substances  T51-T65 Toxic effects of substances chiefly nonmedicinal as to source  T66-T78 Other and unspecified effects of external causes  T79-T79 Certain early complications of trauma  T80-T88 Complications of surgical and medical care, not elsewhere classified  T90-T98 Sequelae of injuries, of poisoning and of other consequences of external causes |
| **XX** External causes of morbidity and mortality | V01-X59 Accidents  X60-X84 Intentional self-harm  X85-Y09 Assault  Y10-Y34 Event of undetermined intent  Y35-Y36 Legal intervention and operations of war  Y40-Y84 Complications of medical and surgical care  Y85-Y89 Sequelae of external causes of morbidity and mortality  Y90-Y98 Supplementary factors related to causes of morbidity and mortality classified elsewhere |
| **XXI** Factors influencing health status and contact with health services | Z00-Z13 Persons encountering health services for examination and investigation  Z20-Z29 Persons with potential health hazards related to communicable diseases  Z30-Z39 Persons encountering health services in circumstances related to reproduction  Z40-Z54 Persons encountering health services for specific procedures and health care  Z55-Z65 Persons with potential health hazards related to socioeconomic and psychosocial circumstances  Z70-Z76 Persons encountering health services in other circumstances  Z80-Z99 Persons with potential health hazards related to family and personal history and certain conditions influencing health status |
| **XXII** Codes for special purposes | U00-U49 Provisional assignment of new diseases of uncertain etiology or emergency use  U82-U85 Resistance to antimicrobial and antineoplastic drugs |
